# A review on the green synthesis of metal (Ag, Cu, and Au) and metal oxide (ZnO, MgO, Co_3_O_4_, and TiO_2_) nanoparticles using plant extracts for developing antimicrobial properties

**DOI:** 10.1039/d5na00037h

**Published:** 2025-03-07

**Authors:** Israt Jahan Lithi, Kazi Imtiaz Ahmed Nakib, A. M. Sarwaruddin Chowdhury, Md. Sahadat Hossain

**Affiliations:** a Department of Applied Chemistry and Chemical Engineering, Faculty of Engineering and Technology, University of Dhaka Dhaka 1000 Bangladesh; b Institute of Glass & Ceramic Research and Testing, Bangladesh Council of Scientific and Industrial Research (BCSIR) Dhaka 1205 Bangladesh saz8455@gmail.com

## Abstract

Green synthesis (GS) is a vital method for producing metal nanoparticles with antimicrobial properties. Unlike traditional methods, green synthesis utilizes natural substances, such as plant extracts, microorganisms, *etc.*, to create nanoparticles. This eco-friendly approach results in non-toxic and biocompatible nanoparticles with superior antimicrobial activity. This paper reviews the prospects of green synthesis of metal nanoparticles of silver (Ag), copper (Cu), gold (Au) and metal oxide nanoparticles of zinc (ZnO), magnesium (MgO), cobalt (Co_3_O_4_), and titanium (TiO_2_) using plant extracts from tissues of leaves, barks, roots, *etc.*, antibacterial mechanisms of metal and metal oxide nanoparticles, and obstacles and factors that need to be considered to overcome the limitations of the green synthesis process. The clean surfaces and minimal chemical residues of these nanoparticles contribute to their effectiveness. Certain metals exhibit enhanced antibacterial properties only in GS methods due to the presence of bioactive compounds from natural reducing agents such as Au and MgO. GS improves TiO_2_ antibacterial properties under visible light, while it would be impossible without UV activation. These nanoparticles have important antimicrobial properties for treating microbial infections and combating antibiotic resistance against bacteria, fungi, and viruses by disrupting microbial membranes, generating ROS, and interfering with DNA and protein synthesis. Nanoscale size and large surface area make them critical for developing advanced antimicrobial treatments. They are effective antibacterial agents for treating infections, suitable in water purification systems, and fostering innovation by creating green, economically viable antibacterial materials. Therefore, green synthesis of metal and metal oxide nanoparticles for antibacterial agents supports several United Nations Sustainable Development Goals (SDGs), including health improvement, sustainability, and innovation.

## Abstract 2

Green synthesis using plant extract is used to tune the antimicrobial property of metals and metal oxides nanoparticles.

## Graphical abstract

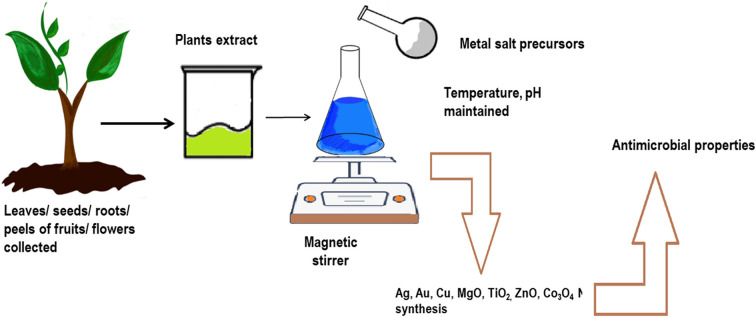

## 1. Introduction

Nanotechnology involves working with materials and synthesizing processes at the nanoscale, with diameters ranging from 1 to 100 nm. Many sectors, including electronics, environmental science, medicine, agriculture, and water management, have gained immense interest in nanoparticles lately.^1–3^ Among the many types of nanomaterials available, the global formation and utilization of metal and metal oxide nanoparticles (NPs) have been expanding rapidly due to their distinctive properties, for example, high surface area to volume ratio and magnetic, catalytic, diminutive, and antimicrobial properties.^4,5^

Nanoparticles are helpful in managing microbial infections since they have some level of antimicrobial activity that kills microorganisms, especially in today's world, where research is being done with a view to generate new antibiotics to fight against the increased resistance of pathogens towards pre-existing antibiotics.^6^

There are two types of methods by which nanoparticles can be produced: the bottom-up and the top-down approach.^7^ The top-down approach breaks down large materials into nanoscale molecules using mechanical or physical processes like lithography, milling, laser ablation, and grinding.^8,9^ However, it can be energy-intensive and less suitable for applications requiring precise control over particle characteristics.^10^ These limit the application of top-down techniques in producing high-quality nanoparticles. The bottom-up approach involves the construction of nanoparticles from atomic or molecular constituents by chemical or biological means. This approach has increased surface control over the appearance and dimension of nanoparticles. Bottom-up methods include chemical reduction, sol–gel, and biological methods.^11^

Traditional nanoparticle production methods require toxic chemicals and harmful organic solvents that cause damage to the environment.^12^ They also need high pressures and temperatures, which cause the cost of production to escalate, while the availability of raw materials is even limited sometimes. Contrarily, green synthesis involves the use of living resources like fungi, bacteria, lichens, or plant extracts, which are safe for the environment and reduce the use of hazardous chemicals.^13^ This bottom-up approach is also non-toxic and cost-efficient.^14^ Green synthesis can easily be scaled up and down in industry for large-scale nanoparticle production or synthesized in the laboratory using green chemistry principles.^15^ Among other biological routes, the plant extract-mediated process to synthesize metal nanoparticles has acquired significant interest due to alkaloids and polyphenols, which were used as capping agents that reduce metallic ion precursors to their nanoparticle forms, thus stabilizing them.^16^

This paper discusses the antimicrobial activities and medical applications of these nanomaterials (gold, copper, zinc oxide, silver, magnesium oxide, cobalt oxide, and titanium dioxide) obtained through green synthesis. These metal NPs have enhanced synergy, resulting in higher antimicrobial efficacy against Gram-positive and Gram-negative bacteria, making them suitable for medical instrument coatings.^17^ They also demonstrate antimicrobial properties even at low concentrations, inhibiting in a dose-dependent manner and being effective against multidrug-resistant strains.^18^ In the case of metal oxides, ZnO exhibits high antibacterial efficacy at low concentrations, effective against both Gram-positive and Gram-negative bacteria, and is pH- and temperature-dependent.^19^ Ti oxide has photocatalytic activity under UV light and produces ROS, which are effective for self-cleaning surfaces but require UV exposure. Despite being biocompatible, MgO is not well known for its strong antibacterial properties. The mechanisms by which nanoparticles show their antimicrobial properties and their applications in the medical and environmental fields are discussed here. This review will contribute to studying these metal and metal oxide nanoparticle addition through a green synthesis process, which has reduced toxicity and improved biocompatibility. The primary objectives of this review are (i) the importance of green synthesis in metal and metal oxide nanoparticles, (ii) the application of these nanoparticles in their respective sectors and their efficiency in the green synthesis method, (iii) green synthesis of metal (Ag, Au, and Cu) and metal oxide (ZnO, MgO, Co_3_O_4_, and TiO_2_) nanoparticles using plant extraction methods and their antibacterial mechanism against microorganisms reviewed from research conducted in recent years, (iv) antibacterial mechanisms of metal and metal oxide nanoparticles, and (v) obstacles of the green synthesis method found in recent research and efficient factors to be considered as recommended by successful research to overcome the limitations of this method.

## 2. Prospects and future of green synthesis

**Fig. 1:**
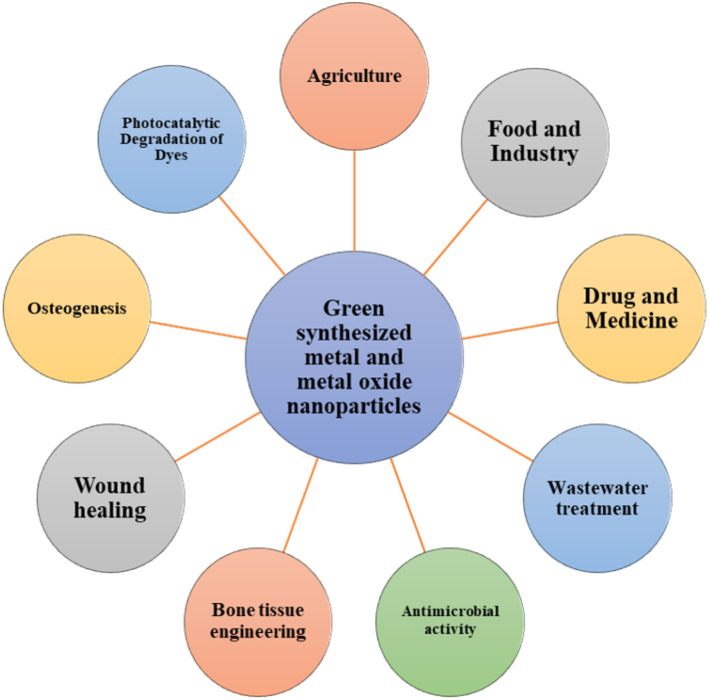
General application of GS metal nanoparticles.^24^

The green synthesis of nanoscale metals is possible, according to studies conducted on a variety of plant materials. Numerous studies on the green synthesis of nanoscale metals have been published in recent years. The green synthesis (GS) of metal nanoparticles (NPs) from microorganisms and plants, along with their uses, are the main topics of this review. Compared to other traditional methods, such as physical and chemical methods, the green synthesis method offers a clean, non-toxic, and environmental friendly way to synthesize metal nanoparticles and agree with the principles of green chemistry.^20^ Nanoparticles have more ROS and antimicrobial properties, primarily because of better physiochemical properties, including the larger surface area, which extends their applications to a wide range of areas like medicine, agriculture, industrial processes, and environmental remediation. Numerous plant materials—such as leaves, fruit extracts, seeds, fruits, bark, *etc.*—as well as microorganisms—such as fungi, actinomycetes, bacteria, algae, *etc*.—have demonstrated the capacity to synthesize different metals and metal oxide nanoparticles. However, plant extracts have phytochemicals present in them (flavonoids, alkaloids, phenolic acids, polyphenols, terpenoids, sugars, and proteins), which act as reducing and stabilizing agents in the formation of metal oxide nanoparticles.^21^ The principal plant metabolites used in the formation of metal nanoparticles are flavonoids, including luteolin and quercetin; ascorbic acid; triterpenes; saponins; reducing sugars, including aldoses and ketones; terpenoids like eugenol; amino acids, including tyrosine and tryptophan; and phytochemicals, including glutathiones, metallothioneins, polyphenols, heterocyclic compounds, carboxyls, amines, water-soluble carbohydrates and ascorbates.^22,23^ Besides antimicrobial activity, metal and metal oxide nanoparticles have a large spectrum of applications (Fig. 1). Since they are highly biocompatible, they can be utilized in agriculture, food, wastewater treatment, photocatalytic degradation of dyes, wound healing, bone tissue engineering, heavy metal detection, and medicinal purposes. These natural capping agents also enhance the biocompatibility of the metal NPs with enhanced antimicrobial properties. By selecting specific plants with unique phytochemical compositions, this plant-mediated synthesizing process can help customize specific properties of the nanoparticles for specific purposes such as water purification or antibacterial coatings. Thus, using plant extracts has proved to be simple and cost-effective.^23^

By comparing general nanoparticles and green synthesized nanoparticles, there are a number of key differences that can be clarified.

### 2.1. Synthesis methods

Regular nanoparticles are typically synthesized using chemical reduction methods (such as sodium borohydride), sonochemistry, laser ablation, or sputter deposition. These methods frequently use hazardous chemicals and energy-intensive processes. Green synthesized nanoparticles are made using biological materials such as plant extracts or microorganisms as reducing agents. This method is more environmentally conscious and sustainable.^25^

### 2.2. Environmental impact

Conventional nanoparticle synthesis, which uses toxic chemicals and solvents, can pollute the environment. Green synthesized NPs minimize the environmental impact by avoiding toxic substances during synthesis.^26^

### 2.3. Cost-effectiveness

Regular nanoparticles are typically more expensive due to the high cost of chemicals used in traditional methods. Green synthesized nanoparticles are generally less expensive because natural extracts are used as reducing agents.

Because of their eco-friendliness, cost-effectiveness, and sustainability, green synthesized metal nanoparticles have a bright future as antibacterial agents. These nanoparticles have demonstrated potent antimicrobial properties against a wide range of pathogens, making them useful in a variety of applications. Green synthesis produces nanoparticles from biological materials such as plants or microorganisms, reducing the need for hazardous chemicals and minimizing the environmental impact. Green metal nanoparticles have shown strong antibacterial activity against both Gram-positive and Gram-negative bacteria. Many green-synthesized nanoparticles are biocompatible,^28^ which is important for medicinal applications. Using natural extracts lowers the production costs compared to traditional chemical methods. Furthermore, these nanoparticles can potentially be used in cancer treatment, as drug delivery systems, and as antiviral agents due to their increased therapeutic drug efficiency.^27^

### 2.4. Future directions

#### 2.4.1. Optimization techniques

More research is needed to optimize the synthesis conditions, such as pH, temperature, and precursor concentration, in order to improve the nanoparticle yield and their properties.^28^

#### 2.4.2. Combination therapies

The possibility of combining green synthesized nanoparticles with conventional antibiotics to improve the antimicrobial efficacy while decreasing antibiotic resistance needs to be explored.^29^

#### 2.4.3. Scalability

Expanding green synthesis processes while remaining cost-effective will be critical for their widespread adoption in industries such as healthcare and food safety. ZnO is used in sunscreens in the cosmetic industry. The production of ZnO nanoparticles through a green synthesis method utilizing plant extracts presents a significant opportunity to integrate the antimicrobial properties inherent in these extracts. This approach is expected to enhance the characteristics of ZnO nanoparticles, making them more suitable for application on sensitive skin. In short, the future of green synthesized metal nanoparticles appears promising due to their ability to combat bacterial infections in a sustainable manner while maintaining their effectiveness and safety.

## 3. Antibacterial applications of lichen

Lichens are emerging as effective biosynthesizers for metal nanoparticles (MNPs) with significant antibacterial properties. This biomass is also useful in perfume production and is used as food, dye, bio-indicators, and traditional medicine.^30^ The green synthesis process involves using lichen extracts to reduce metal ions into nanoparticles, such as silver or iron oxide. These nanoparticles demonstrate antimicrobial activity through mechanisms like ROS generation, which damage bacterial DNA and disrupt cell membranes.^31^ Studies have shown that various lichen species can produce Ag, Cu, TiO_2_, ZnO, and Fe_3_O_4_ nanoparticles of different shapes and sizes, enhancing their potential in antimicrobial applications by leveraging their natural biocompatibility and effectiveness against a range of pathogens.^32,33^ The group of organisms such as algae (photobiont) and many types of fungi (mycobiont) that live together in symbiosis is known as lichens.^34^ Lichens are among the most abundant non-vascular biomass in the world. The biomass in found on tree trunks and branches. Host characteristics and climatic differences affect the degree of colonization and distribution. The mycobionts are known to produce several secondary metabolites, such as usnic acid, atranorin (depside), norstictic acid, stictic acid, chloroatranorin, lecanoric acid, depsone, phenolic acid and salazinic acid, that have antimicrobial, antioxidant and anticarcinogenic activities.^35^ These compounds are insoluble in water and are extracted using organic solvents which might make the extraction process time consuming or require higher temperatures.^36^

### 3.1. Key compounds with antibacterial activity

*Usnic acid*, one of the most studied lichen compounds, is well known for its potent antibacterial activity against Gram-positive bacteria. It prevents RNA and DNA synthesis in these bacteria.

*Vulpinic acid* has strong antibacterial properties against certain Gram-positive bacteria, including *Clavibacter michiganensis* subsp.*michiganensis*.^37^*Lobaric acid* has antibacterial properties comparable to streptomycin against certain Gram-positive bacteria. *Perlatolic acid* shows minimum inhibitory concentration (MIC) values, which indicates potent antibacterial activity.

### 3.2. Mechanism of action^38^

Lichen compounds exert antibacterial effects through several mechanisms, including (1) disruption of bacterial cell membranes, (2) interference with DNA replication, and (3) production of reactive oxygen species (ROS). According to a study conducted by Sharma *et al.*, Himalayan lichen (*Hypotrachyna cirrhata*) has been found to successfully synthesize silver nanocolloids with an average particle size of 11.1 nm to be used in heavy metal sensing, which successfully detected Hg^2+^ and Cu^2+^ with LOD values of 1 and 5 mg L^−1^.^39^ Siddiqi *et al.* synthesized silver nanoparticles using *Usnea longissimi* lichen and found that they are effective as antibacterial agents against Gram positive bacteria, such as *Streptococcus mutans*, *Streptococcus viridans*, *Staphylococcus aureus*, *Streptococcus pyogenes*, *Corynebacterium diphtheriae*, and *Corynebacterium xerosis*, and Gram negative bacteria, such as *Klebsiella pneumoniae*, *Pseudomonas aeruginosa*, and *Escherichia coli*.^40^ In another experiment, Ahmad *et al.* produced spherical ZnO nanoparticles successfully using *Permelia perleta* lichen, which showed antibacterial activity against *E. coli* and also developed photocatalytic dye degradation properties against Eriochrome black-T and acridine orange.^41^ Until now, only *Cetraria islandica* is used successfully to synthesize Ag nanoparticles.^42,43^ Thus, they can be used in many fields of applications, such as in medical devices, as antimicrobial coatings, in wound healing, to disinfect water for purification and biofilm control, as antioxidants, and as antidiabetics.^44,45^

## 4. Metal nanoparticles

### 4.1. Silver

Silver is widely used in the pharmaceutical industry due to its high antimicrobial, antifungal, and anticancer properties.^46^ Recent studies have proved that silver is non-toxic and suitable for killing pathogenic microorganisms.^47^ Silver forms a protective barrier for several pathogens and bacteria like Gram-negative bacteria (*E. coli*), Gram-positive bacteria (*S*. *aureus*), viruses, and fungi.^48^ The reticular structure of the peptidoglycan layer of the cell wall determines the mechanical strength of bacteria and hence results in different amounts of silver permeation into cells.^49^

#### 4.1.1. Green synthesis of silver nanoparticles

**Fig. 2:**
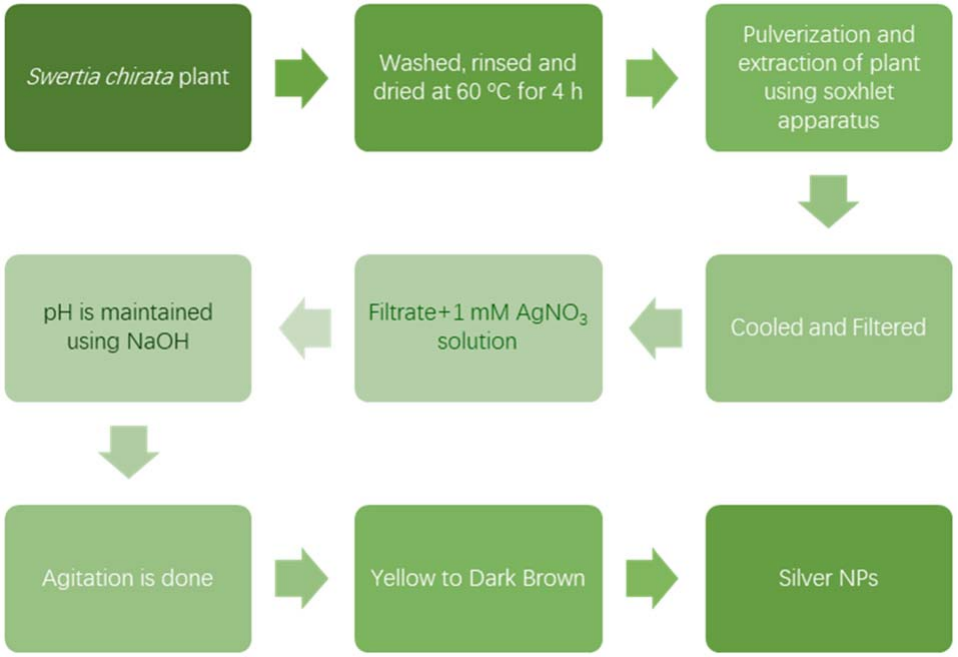
Flow chart of the green synthesis of silver nanoparticles.

According to Shereen *et al.*, the leaves and stem of *Swertia chirata* plant are recommended to be utilized successfully for green synthesis of Ag NPs.^50^ Washed and rinsed plant leaves and stems were dried in an oven at 60 °C for 4 h and then pulverized. They were then extracted in the Soxhlet apparatus using methanol, chloroform, ethanol and distilled water as solvents for 48 h at 60 °C. The resulting extract was then left to cool, filtered and evaporated to complete dryness using a flash evaporator.^50^ 10 mL of the filtrate was added with 90 mL of 1 mM AgNO_3_ solution. Using the basic solution of NaOH, the pH of the solution was immediately brought to 10 and then agitated for 72 h at a rate of 150 rpm until its color changed from yellow to dark brown. Fig. 2 shows the flowchart describing the procedure followed in production of silver nanoparticle by green synthesis process.^51^

Various plant extracts such as *Galega officinalis*, *Psidium guajava*, *Brassica nigra*, and *Ananas comosus* were used to synthesize silver nanoparticles, and it was usually carried out at room temperature (RT) and pH 8-12 (Table 1). *Origanum vulgare* and *Psidium guajava* were found to be effective against a wide range of microbes, including *E. coli*, *Listeria monocytogenes*, *Bacillus cereus*, *Enterococcus faecium*, *S*. *aureus*, *Aeromonas hydrophila*, *Klebsiella* sp., *Salmonella paratyphi*, *Shigella dysenteriae*, *Shigella sonnei*. They kill the bacteria through disruption of microbial membranes, generating ROS and also interaction with sulfur containing compounds. Table 1 depicts the various applications of produced Ag nanoparticles with green method and their potential use as antibacterial agents.

**Table 1:** Formation of Ag nanoparticles from different plant extracts

Raw materials	Plant extract	Reaction conditions	Microbes	Remarks	References
Silver nitrate	*Galega officinalis*	RT, stirred, pH 8–12	*E*. *coli*, *S*. *aureus*, and *Pseudomonas syringae*	Antimicrobial	52
	*Skimmia laureola*	RT	*P*. *aeruginosa*, *E*. *coli*, *Proteus vulgaris*, *S*. *aureus*, and *K*. *pneumoniae*	Antibacterial	53
	*Calophyllum tomentosum*	RT, 1 h	*P*. *aeruginosa*, *S*. *aureus*, *K*. *aerogenes*, and *E*. *coli*	Anti-bacterial and anti-oxidant	54
	*Psidium guajava*	RT, 30 min	*Arthrobacter creatinolyticus*, *Bacillus aryabhattai*, *B*. *subtilis*, *E*. *coli*, *Bacillus megaterium*, and *Alcaligenes faecalis*	Antioxidant and antimicrobial	55
	*Saraca asoca*	Stirring	*E. coli* and *B*. *subtilis*	Anti-bacterial	56
	*Plectranthus amboinicus*	60 °C, 30 min, centrifuged	*E. coli* and *Penicillium* sp*.*	Anti-bacterial	57
	*Brassica nigra*	RT, 1 h, pH 8.5	*Propionibacterium acnes*, *P*. *aeruginosa* and *K*. *pneumoniae*	Anti-bacterial, antifungal, anti-oxidant, and anticancer	58
	*Lavandula angustifolia* Mill.	Boiled for 30 min	*S*. *aureus*, *E*. *coli* and *Candida albicans*	Cytotoxic, anti-oxidant, and antimicrobial	59
	*Ananas comosus* (*L*.)	RT, 24 h, stirred	*L. monocytogenes*, *B*. *cereus*, *E. faecium*, and *S*. *aureus*	Anti-oxidant, anti-bacterial, antidiabetic and cytotoxic	60
	*Prosopis farcta*	RT, 1 h, centrifuged	*S*. *aureus*, *B*. *subtilis*, *P*. *aeruginosa*, and *E*. *coli*	Antibacterial	61
	*Olea europaea*	RT, 2 min, stirred	*S*. *aureus*, *P*. *aeruginosa*, and *E*. *coli*	Antibacterial	62
	*Azadirachta indica*	RT	*E*. *coli*, *P*. *aeruginosa*, and *B*. *subtilis*	Antimicrobial and antiproliferative	63
	*Persea americana*	RT, 5 h, stirred	*E. coli*	Antimicrobial	64
	*Phlomis*	RT	*E*. *coli*, *Salmonella typhimurium*, *S*. *aureus*, and *B*. *cereus*	Antibacterial	65
	*Salvia spinosa*	RT, 6 h, stirred	*B*. *subtilis*, *Bacillus vallismortis*, and *E*. *coli*	Antibacterial	66
	*Artocarpus heterophyllus* Lam.	121 °C, 5 min, 15 psi in autoclave	*B*. *cereus*, *B*. *subtilis*, *S*. *aureus*, *P*. *aeruginosa*, and *P*. *vulgaris*	Antimicrobial	67
	*Camellia sinensis*	50 °C, 30 min	*Klebsiella* sp. and *S*. *aureus*	Antibacterial	68
	*Aloe vera*	RT	*E*. *coli*, *P*. *aeruginosa*, *Enterobacter* sp., and *S*. *aureus*	Antimicrobial	69
	*Boerhaviadiffusa*	RT, 24 h	*A*. *hydrophila*, *Pseudomonas fluorescens*, and *Flavobacterium branchiophilum*	Antibacterial	70
	*Calliandra haematocephala*	80 °C, 10 min	*E. coli*	Antibacterial	71
	*Origanum vulgare*	60–90 °C, 10 min	*A*. *hydrophila*, *Bacillus* sp., *E*. *coli*, *Klebsiella* sp., *S*. *paratyphi*, *S*. *dysenteriae*, and *S*. *sonnei*	Antibacterial and cytotoxic	72
	*Rosmarinus officinalis*	RT, stirred	*S*. *aureus*, *B*. *subtilis*, *E*. *coli*, *P*. *aeruginosa*, *C*. *albicans*, and *Aspergillus oryzae*	Antibacterial and antifungal	73
	*Alternanthera dentata*	RT, 10 min	*E*. *coli*, *P*. *aeruginosa*, *K*. *pneumoniae*, and *Enterococcus faecalis*	Antibacterial	74
	*Berberis vulgaris*	Warm solution	*S*. *aureus*, *P*. *aeruginosa*, *L*. *monocytogenes*, *E*. *coli*, and *Salmonella enterica*	Antibacterial	75
	*Ficus benghalensis*	RT	*P*. *aeruginosa*, *Vibrio cholerae*, *B*. *subtilis*, and *E*. *coli*	Antiproliferative and antimicrobial	63
	*Pistacia atlantica*	80 °C, stirred	*P*. *aeruginosa*, *S*. *aureus*, *S*. *pyogenes*, *E*. *coli*, *S*. *paratyphi B*, and *K*. *pneumoniae*	Antibacterial	76
	*Carissa carandas* L.	RT	*S*. *typhimurium*, *E*. *faecalis*, *Shigella flexneri*, *Citrobacter* spp., and *Gonococci* spp.	Antioxidant and antimicrobial	77
	*Lysiloma acapulcensis*	RT, 2 min, incubated	*E*. *coli*, *P*. *aeruginosa*, *S*. *aureus*, and *C*. *albicans*	Antimicrobial and cytotoxic	78
	*Zephyranthesrosea*	RT, pH 11	*E*. *coli*, *S*. *aureus*, and *S*. *mutans*	Anti-bacterial, anti-oxidant, anti-inflammatory, and anti- diabetic	79

#### 4.1.2. Antimicrobial properties and mechanism of silver nanoparticles

Silver nanoparticles damage the bacterial cell wall by causing DNA damage, preventing the bacteria from multiplying.^80^ It can physically bind to the exterior of microbial cells because of their small dimensions and high ratio of surface to volume. It penetrates the lipid bilayer of microbial cell membranes and disrupts the integrity of the lipid bilayer structure.^47^ This disruption leads to holes in cell membrane and causes cellular content leakage, leading to cell death. Studies also show that silver nanoparticles interacting with the integral membrane proteins, including transporters and ion channels, cause denaturation of the cell. Silver nanoparticles attached to sulfur atoms in enzymes and sulfhydryl protein groups are found in the bacterial cell surface.^81^

Some studies suggest that silver nanoparticles induce programmed cell death in microbial cells.^82^ This process is known as apoptosis. Silver nanoparticles produce reactive oxygen species (ROS) within microbial cells, mainly in the mitochondria. Wang *et al.* suggest that endoplasmic reticulum is highly prone to oxidative damage.^83^ ROS include superoxide radicals like hydroxyl radicals and hydrogen peroxide that cause oxidative stress, DNA damage and mitochondrial dysfunction. Improper ER function causes cell damage and apoptosis,^84^ resulting from damage or reduced disulfide bond formation, inhibition of protein glycosylation, or deficiency of calcium from the ER lumen.^82^ Silver nanoparticles lead to inhibition of enzymatic activity by interacting with metal-binding sites within microbial enzymes, preventing substrate binding. This causes denaturation or loss of enzyme activity. It also alters the pH and temperature stability of the enzymes.^85^ From Table 1, it can be seen that most Ag NPs are ideally synthesized at room temperature (RT).

Hence, researchers are encouraged to explore environmentally friendly and effective methods for producing silver nanoparticles. Among various methods, using microalgae such as seaweeds has proven successful in producing silver nanoparticles.^86^

It has firmly been established that the performance and applicability of silver nanoparticles rely on their shape, composition, and surface chemistry. For instance, brown seaweed *Padina tetrastromatica* produces spherical nanoparticles with a diameter of 14 nm, exhibiting excellent anti-bacterial properties.^87^ Similarly, *Trichoderma viride* and *Chaeroceros calcitrans* are also known to form silver nanoparticles with potent bactericidal activity.^88^*Ecklonia cava*, a brown alga found mainly in Korea, Japan, and China, produces nanoparticles with anti-oxidant, anticancer, and antimicrobial activities.^88^

### 4.2. Gold

Gold has historically been utilized by ancient societies for jewellery, adornments, cosmetics, currency, and medical purposes.^89^ Also, it is seen that during the Middle Ages, people used to drink gold and utilised it for many diseases like arthritis, tumours, heart diseases, epilepsy, dysentery, and many more.^90,91^ Gold nanoparticles have been shown to be very useful as a therapeutic substance in the treatment of tumours.^92^

#### 4.2.1. Green synthesis of gold nanoparticles

To synthesize gold nanoparticles, *Capsicum annum* is widely selected. According to Patil *et al.*, dried red chilies were collected and powdered, and 5 : 100 DI water was made and boiled.^93^ Filtration was done further to remove any remaining residue. The filtrate was added to 10 mL 1 mM HAuCl_4_, and the temperature was brought to 90 °C. The mixture was stirred until the colour turned dark violet from light yellow. It was then cooled and spun at 10 000 rpm for 30 min. The precipitate was rinsed with DI water and stored at 4 °C.^93^

Plant extracts like *Croton caudatus*, *O. europaea*, *Moringa oleifera*, and *Mangifera indica* are most commonly used. The synthesis temperature usually ranges from RT to 75 °C, and the incubation time ranges from minutes to hours. *M. indica* has shown effectiveness against the largest group of microbes such as *B*. *cereus*, *B*. *subtilis*, *S*. *aureus*, *Corallium rubrum*, *E*. *coli*, *P*. *aeruginosa*, *K*. *pneumoniae*, *S*. *typhimurium*, *Cryptococcus neoformans*, *C*. *albicans*, and *Candida glabrata*, proving that gold nanoparticles have excellent antimicrobial, antifungal, antioxidant and anticancer activities. Table 2 illustrates the various applications of gold nanoparticles produced using a green method and their potential use as antibacterial agents.

**Table 2:** Formation of gold nanoparticles from different plant extracts

Raw materials	Plant extract	Reaction conditions	Microbes	Remarks	References
Chloroauric acid	*CrotoncaudatusGeiseler*	RT	*E. coli*	Cytotoxic, antimicrobial, antioxidant, and antifungal	94
	*Mammea suriga*	30 °C, 24 h, stirred	*E*. *coli*, *S*. *aureus*, *P*. *aeruginosa*, and *B*. *subtilis*	Antibacterial	95
	*Olea europaea* and *Acacia nilotica*	40 °C, 15 min, stirred at 1000 rpm	*S*. *aureus*, *B*. *subtilis*, *E*. *coli*, *K*. *pneumoniae*, and *P*. *aeruginosa*	Antibacterial and cytotoxic	96
	*Citrus maxima*	RT, stirred	*S*. *aureus* and *E*. *coli*	Catalytic and antibacterial	97
	*Ziziphus zizyphus*	RT	*E*. *coli* and *C*. *albicans*	Antibacterial and antifungal	98
	*Couroupita guianensis*	RT, 5 min	HL-60 cells	Cytotoxic and anticancer	99
	*Simarouba glauca*	RT, 15 min	*S*. *aureus*, *S*. *mutans*, *B*. *subtilis*, *E*. *coli*, *P*. *vulgaris*, and *K*. *pneumoniae*	Antibacterial	100
	*Zingiberofficinale*	60 °C, 60 min, stirred, pH = 8	*P*. *aeruginosa*, *E*. *coli*, *B*. *subtilis*, *S*. *aureus*, *C*. *albicans*, and *Aspergillusbrasiliensis*	Antibacterial and antifungal	101
	*Pterocarpus santalinus* L.	28 °C, pH = 7.4	*S*. *aureus* and *P*. *aeruginosa*	Antimicrobial	102
	Thyme	RT, stirred, 30 min	*E*. *coli*, *P*. *aeruginosa*, *S*. *aureus*, and *B*. *subtilis*	Antioxidant, antimicrobial, and cytotoxic	103
	*Kaempferiaparviflora*	RT, 30 min, stirred at 200 rpm	*E*. *coli*, *P*. *aeruginosa*, *S*. *aureus*, and *B*. *subtilis*	Catalytic, antioxidant, and antimicrobial	104
	*Garcinia kola*	RT, 5 h, stirred	*P*. *aeruginosa*, *E*. *coli*, *S*. *aureus*, and *B*. *cereus*	Antibacterial	105
	*Moringa oleifera*	75°C, stirred	*S*. *aureus*, *E*. *coli*, and *Enterococcus sp.*	Cytotoxic, antioxidant, antibacterial, and photo-catalytic	106
	*Caulerpa racemosa*	RT, 24 h, stirred	*Aeromonasveronii* and *Streptococcus agalactiae*	Anticancer and antibacterial	107
	*Lannea discolor*	25 °C, 1 h	*S*. *aureus*, *P*. *aeruginosa*, *K*. *pneumoniae*, and *E*. *coli*	Antibacterial	108
	*Trianthema decandra*	RT, dark place	*E*. *coli*, *Streptococcus faecalis*, *P*. *aeruginosa*, *B*. *subtilis*, and *S*. *aureus*	Antimicrobial	109
	*Areca catechu*	300 K, pH 6, 5 min, stirred	*E*. *coli*, *Enterobacter* sp., *K*. *pneumoniae*, *P*. *aeruginosa*, and *S*. *aureus*	Anticancer, antibacterial, antioxidant, and catalyst	110
	*Parthenium hysterophorus*	70 °C, 60 min, stirred	*S*. *aureus*, *E*. *coli*, *E*. *faecalis*, and *S*. *enterica*	Antibacterial and antifungal	111
	*Bauhinia purpurea*	RT, microwave irradiation	*S*. *aureus*, *P*. *aeruginosa*, *B*. *subtilis*, *Aspergillus flavus*, and *E*. *coli*	Anticancer, antimicrobial, antioxidant, and catalytic	112
	*Jatrophaintergerrima*	RT	*B*. *subtilis*, *S*. *aureus*, *E*. *coli*, and *K*. *pneumoniae*	Antibacterial	113
	*Nepenthes khasiana*	RT, 3 h, stirred	*Bacillus* sp., *E*. *coli*, *C*. *albicans*, and *Aspergillus niger*	Antimicrobial	114
	*Mangifera indica*	RT, 24 h, dark	*B*. *cereus*, *B*. *subtilis*, *S*. *aureus*, *C*. *rubrum*, *E*. *coli*, *P*. *aeruginosa*, *K*. *pneumoniae*, *S*. *typhimurium*, *C*. *neoformans*, *C*. *albicans*, and *C*. *glabrata*	Antimicrobial, antioxidant, and anticancer	115
	*Allium cepa* L.	RT, 24 h, stirred	*B*. *cereus*, *E*. *coli*, *L*. *monocytogenes*, *S*. *aureus*, *S*. *typhimurium*, and *C*. *albicans*	Antibacterial, antifungal, and antioxidant	116
	*Eclipta alba*	90 °C, 20 min	*B*. *subtilis*, *E*. *coli*, *S*. *aureus*, and *P*. *aeruginosa*	Antibacterial and antidiabetic	117

#### 4.2.2. Antimicrobial properties and mechanism of gold nanoparticles

Having a small size and high optical and inert properties, gold nanoparticles can accumulate in tumours and hence help in thermal treatment procedures.^118^ They are effective delivery systems for anticancer medications, reducing both the duration of treatment and its adverse effects,^119^ having high chemical inertness, size and shape control, surface oxidation resistance, and relative stability.^120^ Also, gold nanoparticles have special properties, such as surface plasmon resonance (SPR), making them valuable for the treatment of cancer, imaging of tissues, and delivery of drugs and biosensors.^121,122^ A study by Chauhan *et al.* proved the potential of gold nanoparticles for the detection of liver cancer.^123^ The study proved that due to the antibiotic properties of gold nanoparticles, they can successfully differentiate between cancerous cells and normal cells. Research has found that gold nanoparticles can destroy Gram-negative bacteria faster than Gram-positive bacteria. They show different antimicrobial activities with different bacterial cell wall compositions. Compared to Gram negative bacteria, Gram positive bacteria have a thicker peptidoglycan coating of linear polysaccharides cross-linked by peptides, making penetration more difficult for nanoparticles. Recent research has proved that gold nanoparticles produced from natural sources have potent anti-inflammatory and cytotoxic effects against cancer cells.^121^ They are also effective against diabetic management and have environmental uses like the catalytic breakdown of organic dyes and antimicrobial properties. Khalil *et al.* proved its antioxidant properties in a research article using olive leaf extract to synthesize gold nanoparticles.^124^ The nanoparticles synthesized from plant extract can also be used in agriculture due to their low toxicity.^125^ They are used to target specific areas in plants and inject nutrients or genes to genetically modify any plant to increase its nutrition level.

Gold nanoparticle synthesis using plant extracts has shown to be beneficial in producing nanoparticles with a variety of shapes and sizes influenced by the specific biological entities involved and reacting conditions like reaction time, temperature, reactant concentration and pH.^90^ For example, adding an extract of *Stachys lavadulifolia Vahl* to a gold ion solution can produce hexagonal, spherical, and triangular shaped particles.^126^ The quantity of extract and reaction temperature are also important under different conditions that lead to different-sized nanoparticles.

Polyphenols in plant extracts cause the reduction of Au to form nanoparticles.^91^ Also, green synthesis of gold nanoparticles means no external stabilizing agent is required, and it is a renewable process with high availability of cheap raw materials for reaction conditions.^127^

### 4.3. Copper

Recent studies on the bactericidal effects of copper nanoparticles showed that copper nanoparticles exhibited better anti-bacterial activity than silver nanoparticles.^128^ Also, copper nanoparticles were preferred over silver nanoparticles since copper is cheaper than silver, has higher chemical and physical stability, and mixes easily with polymers.^129^ Copper nanoparticles have many uses in numerous fields, due to their desirable optical, magnetic, and electrical characteristics, and are used in nanoparticle devices.^130^ Copper has ultraviolet sensitivity, catalytic activity, and electrical conductivity; is malleable; possesses anti-bacterial and antifungal activities; has high thermal and corrosion resistance.^131,132^ Copper nanoparticles have been used in water treatment, as antimicrobial coatings for medical equipment, and in heat transfer systems.^133^

#### 4.3.1. Green synthesis of copper nanoparticles

Following the procedure proposed by Amer *et al.,* fresh *Citrus limon* fruits were washed thoroughly with distilled and deionised (DI) water, and the required plant extract was made through heating the cut pieces in 100 mL of deionised water and filtering the solution.^134^ The extract was added with 4 g copper sulfate pentahydrate (CuSO_4_·5H_2_O) at 27 °C for 4 h. The reaction was completed within 10 min as the color of the extract changed from blue to brown. Later, at 3000 rpm, it was centrifuged for 10 min. At 85 °C, it was dried for 4 h.^134^

Many plant extracts such as *Punica granatum, Citrus limon*, *Ocimum sanctum*, *Allium saralicum*, *Syzygium aromaticum*, and *Aloe vera* are commonly used to synthesize copper nanoparticles. The reaction temperature ranges from RT to 90 °C under different pH conditions. These nanoparticles have been found to have strong antibacterial action against *S*. *aureus*, *P*. *aeruginosa*, *E*. *coli*, and *K*. *pneumoniae*, as shown in Table 3. Table 3 shows various applications of produced Cu nanoparticles using the green method and their potential use as antibacterial agents.

**Table 3:** Formation of copper nanoparticles from different plant extracts

Raw materials	Plant extract	Reaction conditions	Microbes	Remarks	References
Copper(ii) sulfate	*Punica granatum*	80 °C, 4 h stirring	*Micrococcus luteus, P*. *aeruginosa*, *S*. *enterica*, and *Enterobacteraerogenes*	Anti-bacterial	135
	*Alstonia scholaris*	RT	*E*. *coli* and *S*. *aureus*	Antimicrobial	136
	*Lannea discolor*	25 °C, 1 h	*P*. *aeruginosa*, *S*. *aureus*, *E*. *coli*, and *K*. *pneumoniae*	Antibacterial	108
	*Sesbania aculeata*	RT, 24 h, stirred	*Phoma destructiva* and *Curvularialunata*	Antimicrobial	137
	*Celastrus paniculatus*	RT, pH-7	*Fusariumoxysporum*	Photocatalytic and antifungal	138
	*Cedrusdeodara*	RT, stirred, kept in dark for 5 days	*S*. *aureus* and *E*. *coli*	Antibacterial	139
	*Allium saralicum*	RT, 1 h, stirred, pH-12	*C*. *albicans*, *C*. *glabrata*, *Candida krusei*, *Candidaguilliermondii*, *P*. *aeruginosa*, *E*. *coli*, *B*. *subtilis*, and *S*. *aureus*	Antioxidant, cytotoxic, antifungal, and antibacterial	140
	*Ziziphus spina-christi* (L.)	RT	*S*. *aureus* and *E*. *coli*	Antibacterial and dye adsorbent	141
	*Ocimum sanctum*	RT, stirred, 1 h, sunlight	*E*. *coli*, *K*. *pneumoniae*, *E*. *faecalis*, and *S*. *aureus*	Antibacterial, cytotoxic, and antioxidant	142
	*Nigella sativa*	80 °C, stirred at 150 rpm	*S*. *aureus*, *K*. *pneumoniae*, *E*. *coli*, and *P*. *aeruginosa*	Antibacterial, lipase, amylase, and glucose assay activity	143
	*Grewia asiatica* L.	70 °C, 24 h, stirred at 140 rpm	*A. niger*, *A*. *oryzae*, *E*. *coli*, and *B*. *subtilis*	Antibacterial, antifungal, larvicidal, and anti-termite	144
	*Azadirachta indica*	80 °C, 1 h, stirred at 700 rpm	*Proteus mirabilis*, *K*. *pneumoniae*, and *S*. *aureus*	Antibacterial	145
	*Cissus vitiginea*	RT, stirred	*Enterococcus* sp., *Klebsiella* sp., *Proteus* sp., and *E*. *coli*	Antioxidant and antibacterial	146
	*Citrus limon*	27 °C, 4 h, stirred	*E*. *coli* and *S*. *aureus*	Antibacterial	134
Copper nitrate	*Hagenia abyssinica (Brace) JF. Gmel*	RT, 24 h, centrifuged	*S*. *aureus*, *P*. *aeruginosa*, *B*. *subtilis*, and *E*. *coli*	Antimicrobial and anti-oxidant	128
	*Withania somnifera*	55 °C, 4 h, stirred	*E*. *coli* and *S*. *aureus*	Antibacterial and antioxidant	147
	*Cinnamomumzelanicum*	65 °C, 24 h, stirred	Lung carcinoma cell line	Cytotoxic and antioxidant	148
	*Garcinia mangosteen*	70 °C, 1 h	*S*. *aureus* and *E*. *coli*	Antibacterial	149
	*Eryngium caucasicum* Trautv.	60 °C, 24 h, stirred	*S*. *typhimurium*, *B*. *cereus*, *E*. *coli*, and *S. aureus*	Antimicrobial	150
Copper(ii) chloride	Green tea	100 °C, 3 h, refluxed	*S*. *aureus*, *B*. *subtilis*, *P*. *aeruginosa*, and *E*. *coli*	Antibacterial	151
	*Cardiospermum halicacabum*	80 °C, 1 h, stirred	*P*. *aeruginosa*, *E*. *coli*, and *S*. *aureus*	Antibacterial	152
	*Ageratum houstonianum* Mill.	Erlenmeyer flask, RT, 24 h, stirred	*E. coli*	Photocatalytic and anti-bacterial	153
Copper acetate	*Syzygium aromaticum*	RT, 15 min, stirred	*Staphylococcus* sp.*Pseudomonas* sp. *E*. *coli*, and *Bacillus* sp.	Antimicrobial and antifungal	154
	*Aloe vera*	70 °C, 15 min, stirred, pH-11.5	*B*. *subtilis*, *S*. *aureus*, and *E*. *coli*	Antimicrobial	155
	*Kigelia africana*	RT, 3 h, stirred	*Salmonella typhi*, *E*. *coli*, *P*. *aeruginosa*, and *S*. *aureus*	Antimicrobial	156

#### 4.3.2. Antimicrobial properties and mechanism of copper nanoparticles

The antimicrobial properties depend on the nanoparticles' concentration, stability, and size, which are very important for biomedical and pharmaceutical applications.^150^ According to Rajesh *et al.*, copper nanoparticles have shown excellent antibacterial activity against *Bacillus* spp. and excellent antifungal activity against *Penicillium* spp.^154^ Due to the usage of more and more antibiotics in our body, we build antibacterial resistance, which may occur in various ways, like changes in target sites of antimicrobial agents, reduced intracellular accumulation, or enzyme modification due to resistant genes.^157^ Thus, it has been a challenge to find and improve antibacterial agents so that they are effective in killing the bacteria, both Gram negative and Gram positive, including *Pasteurella multocida*, *K. pneumoniae*, *Streptococcus pneumoniae*, and *S. typhi*, and hence the use of nanoparticles for antibacterial properties has evolved.^158^

Copper nanoparticles release Cu^2+^ ions, which bind to the bacteria cell wall and neutralize it; hence, the penetrability of the membrane increases, and cell lysis occurs. It also damages the cell membrane and causes the cellular components to leak out, which in turn kills the cell.^159^ A study by Kumar *et al.* stated about lipase inhibition activity, showing that copper nanoparticles synthesized from the plant extract of *Saussurea lappa* also have anti-obesity and anti-diabetic activities due to the flavonoid present in it.^160^

One of the most prevalent elements on Earth is copper. It is found in more than 30 types of protein, and many enzymes in our body need copper to function properly. Both physical and chemical methods have been used to form Cu nanoparticles, including microemulsion-based and chemical reduction methods.^161^ Formation of Cu nanoparticles with the green method is very successful for their environment-friendly nature, cost-effectiveness, availability, and non-hazardous by-products.^162^ Also, it is a sustainable and rapid process with a simple reaction setup and very mild reaction conditions.

## 5. Metal oxide nanoparticles

### 5.1. Zinc oxide

ZnO nanoparticles have also gained much interest in medicine, catalysis, electronics, and optics because of their outstanding electrical and antimicrobial characteristics.^163,164^ ZnO nanoparticles have been incorporated in sunscreens and cosmetic lotions because of their property to absorb and reflect UV radiation, which protects from the danger of sunburn and skin cancer.^165^ It is also effective as an anti-bacterial agent against a broad spectrum of bacteria.^166^ Therefore, green synthesised ZnO nanoparticles are essential as they are eco-friendly, can replace synthetic chemicals, and have very effective therapeutic and antimicrobial actions.^167^

#### 5.1.1. Green synthesis of ZnO nanoparticles

**Fig. 3:**
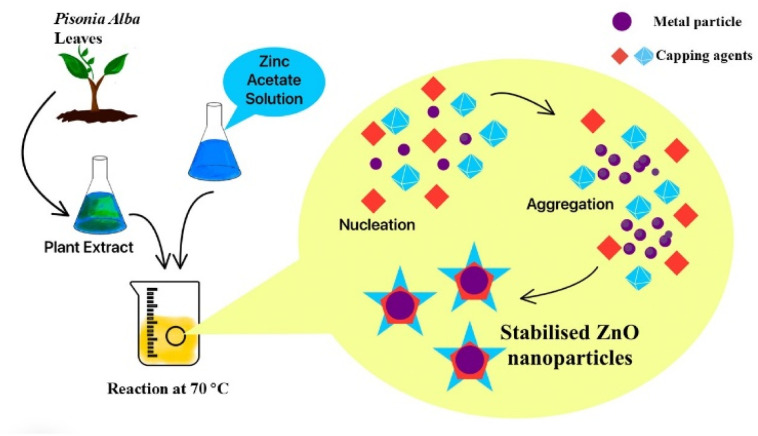
Schematic presentation of the synthesis mechanism of ZnO nanoparticles using plant extracts.

*Pisonia alba* leaves were gathered and rinsed carefully with DI water, as demonstrated by MuthuKathija *et al.*^168^ 10 g of powdered leaves was put in 50 mL of deionised Milli Q water^169^ to make the leaf extract. Then, the prepared extract was cooled, filtered, and stored at 4 °C. 20 mL of the extract solution was added with 50 mL of zinc acetate solution, and at 70 °C, the mixture was swirled continually for 2 h. At 6000 rpm, it was centrifuged for 15 min, the precipitate was then collected after filtration, rinsed, and dried at 80 °C. Fig. 3 shows the synthesis mechanism of ZnO nanoparticles when *Pisonia Alba* leaves are used.^170^

*Aloe vera*, *Citrus aurantifolia*, *Terminalia chebula*, *Azadirachta indica*, *Elaeagnus angustifolia*, and many other plant extracts are used to form ZnO nanoparticles. *Atalantia monophyla* and *Elaeagnus angustifolia* are found to have antimicrobial properties against the maximum number of microbes such as *Leishmania tropica*, *Mucor racemosus*, *A*. *niger*, *Fusarium solani*, *C*. *albicans*, *A*. *flavus*, *S*. *aureus*, *E*. *coli*, *P*. *aeruginosa*, *K*. *pneumoniae*, *B*. *subtilis*, and *B*. *cereus* according to Table 4. The condition temperature ranges from 50–80 °C. These nanoparticles are found to have broad-spectrum antibacterial effects, including both Gram-positive and Gram-negative bacteria, fungi and also have antioxidant and anticancer properties. Table 4 illustrates the various applications of produced ZnO nanoparticles using a green method and their potential use as antibacterial agents.

**Table 4:** Formation of ZnO nanoparticles from different plant extracts

Raw materials	Plant extract	Reaction conditions	Microbes	Remarks	References
Zinc nitrate	*Aloe vera*	60 °C, stirred	*E*. *coli*, *K*. *pneumoniae*, *P*. *aeruginosa*, *B*. *subtilis*, *S*. *aureus*, and *S*. *typhi*	Antimicrobial and anti-bacterial	171
	*Cymbopogancitratus*	RT, 5 min, stirring	*E*. *coli* and *S*. *aureus*	Anti-bacterial	172
	*Coleus amboinicus*	60–80 °C, stirred	*S*. *typhi*, *K*. *pneumoniae*, *E*. *coli*, *B*. *cereus*, and *S*. *aureus*	Antimicrobial	173
	*Azadirachta indica*	80 °C, stirred	*B*. *subtilis*, *P*. *mirabilis*, *S*. *aureus*, *P*. *aeruginosa*, and *E*. *coli*	Antimicrobial	174
	*Solanum nigrum*	60–80 °C	*S*. *aureus*, *S*. *paratyphi*, *V. cholerae* and *E*. *coli*	Anti-bacterial	175
	*Citrus aurantifolia*	65–70 °C, stirring	*E*. *coli*, *A*. *niger*, and *T*. *viride*	Antimicrobial	176
	*Terminalia chebula*	RT, 30 min	*S*. *enterica*, *S*. *aureus*, and *E*. *coli*	Anti-bacterial	177
	*Justicia adhatoda*	60 °C, 1 h, stirred	*S*. *aureus*, *K*. *pneumoniae*, *E*. *coli*, and *S*. *pneumoniae*	Antimicrobial	178
	*Punica granatum*	60 °C, 2 h, stirred	*S*. *aureus*, *E*. *coli*, *S*. *pneumoniae*, *B*. *cereus*, *Moraxella catarrhalis*, *A*. *hydrophila*, *S*. enterica subsp. *diarizonae*, and *E*. *faecalis*	Antibacterial	179
	*Elaeagnus angustifolia*	60 °C, 2 h	*L*. *tropica*, *M*. *racemosus*, *A*. *niger*, *F*. *solani*, *C*. *albicans*, *A*. *flavus*, *S*. *aureus*, *E*. *coli*, *P*. *aeruginosa*, *K*. *pneumoniae*, and *B*. *subtilis*	Antileishmanial, antioxidant, anticancer, and antifungal	180
	*Beta vulgaris*	60 °C	*E*. *coli* and *S*. *aureus*	Antibacterial and antifungal	181
	*Tabernaemontana divaricata*	80 °C	*S*. *paratyphi*, *E*. *coli*, and *S*. *aureus*	Antimicrobial and photocatalytic	182
	*Acacia caesia*	65 °C	*E*. *coli*, *C.albicans*, *S*. *aureus*, and *A*. *niger*	Antimicrobial, antifungal, and anti-inflammatory	183
	*Tectona grandis* (*L*.)	60–90 °C	*S*. *aureus*, *S*. *paratyphi*, *B*. *subtilis*, and *E*. *coli*	Anti-bacterial, anti-arthritic, and anti-oxidant	184
Zinc(ii) acetate	*Eucalyptus lanceolatus*	65 °C, 5 h	*E*. *coli*, *F. solani*, *S*. *aureus*, *P*. *multocida*, *B*. *subtilis*, *Aspergillus parasiticus*, and *A*. *niger*	Antimicrobial, anti-bacterial, and antifungal	185
	*Hibiscussubdariffa*	50 °C, 30 min, stirred	*E*. *coli* and *S*. *aureus*	Antibacterial and anti-diabetic	186
	*Dilleniaindica* and *Mikania micrantha*	60 °C, 2 h, stirred	*S*. *typhi*, *V*. *cholerae*, *S*. *aureus*, and *E*. *coli*	Antibacterial and photocatalytic	187
	*Passiflora caerulea* L.	RT, stirred, 3 h	*E*. *coli*, *Streptococcus* sp., *Enterococcus* sp., and *Klebsiella* sp.	Antibacterial	188
	*Atalantia monophylla*	60 °C, 2 h, stirred	*B*. *Subtilis*, *B*. *cereus*, *S*. *aureus*, *E*. *coli*, *P*. *aeruginosa*, *K*. *pneumoniae*, *C*. *albicans*, and *A*. *niger*	Antimicrobial	189
	*Litchi chinensis*	70 °C, 3 h, stirred at 180 rpm	*P*. *aeruginosa*, *B*. *subtilis*, and *E*. *coli*	Antibacterial and dye removal	190
	*Musa paradisiaca*	85 °C, 2 h, stirred, pH = 12	*S*. *enterica*, *K*. *pneumoniae*, *B*. *cereus*, and *S*. *aureus*	Antimicrobial	191
	*Polyalthia longifolia*	60 °C, 2 h, stirred	*F. oxysporum* f. sp. *ciceris*	Antifungal and photocatalytic	192
	*Cassiaauriculatacan*	RT, 2 h, stirred	*B. subtilis*, *K*. *pneumoniae*, *P*. *aeruginosa*, and *P*. *mirabilis*	Antibacterial	193
Zinc oxide	*Olea europaea*	Stirred at 100 rpm for 4 h	Xoo strain GZ 003	Antibacterial	194

#### 5.1.2. Antimicrobial properties and mechanism of zinc oxide nanoparticles

ZnO nanoparticles have extensive biomedical applications, including anticancer, antimicrobial, antidiabetic, and drug delivery systems.^195^ Most of the current research demonstrates that ZnO nanoparticles can exhibit antibacterial properties by eliminating bacterial cells solely by forming ROS.^196^ It can produce superoxide radicals (O_2_^−^), hydrogen peroxide (H_2_O_2_), and hydroxyl radicals (˙OH)^197^ when exposed to UV radiation or enough moisture.^198^ These then diffuse into the infected cells, induce oxidative stress,^199^ damage the DNA, and ultimately destroy the cell.^200^ Also, ZnO nanoparticles have the potential to destroy cells upon contact by disrupting the cell membrane, ultimately leading to death of cells through a process called apoptosis.^201^ The bacterial cell wall has a peptidoglycan layer that encompasses the cell membrane and holds the cytoplasm's osmotic pressure, thus giving the cell its shape. Gram-negative bacteria possess two cell membranes, an outer membrane and a plasma membrane. Gram negative bacterial cell wall is characterized by a 7–8 nm narrow peptidoglycan layer.^202^ In general, the bacterial cell wall caries a negative charge. Gram positive bacteria have a single membrane made of a multilayered peptidoglycan polymer, with a cell wall of 20–80 nm thickness.^203^

For the formation of ZnO nanoparticles, chemical, biological, and physical methods were used; they include hydrothermal method, microwave-based synthesis, laser ablation, sol–gel method, *etc.*^204–206^ These methods, however, present several problems, such as the need for high pressures or temperatures, high cost, and the time-consuming involvement of harmful reagents that are detrimental to the surroundings and health of those who use them.^207^ Hence, there have been efforts to synthesize ZnO nanoparticles through green, reliable, cost-effective, energy-efficient, and sustainable methods. Biosynthesized nanoparticles are shown to have more catalytic activity and allow large-scale production of nanoparticles.^208^ The process of creating green nanoparticles involves oxidizing or reducing metallic ions by an organic group. This is a bottom-up approach. The mechanism of using extracts of plants involves using the active molecule present in plants, which acts as a capping and a reducing agent.^209^ These active molecules, known as phytochemicals, reduce metal ions to produce nanoparticles and stabilise them by attaching to their surfaces.^210^ Thus, they help to control the dimension, dispersity, and appearance of nanoparticles, which is essential for various uses.^211^

Different plant extracts produce nanoparticles with different properties. For example, ZnO nanoparticles produced from *Eucalyptus lanceolatus* have anti-bacterial and antifungal properties,^212^ whereas *Acacia caesia* produce ZnO nanoparticles with antifungal, antimicrobial, and anti-inflammatory action.^183^ They also affect the appearance and dimensions of nanoparticles produced. Hence, the choice of the plant extract to use in synthesizing ZnO nanoparticles with the desired properties becomes essential. Thus, the process of selecting the plant extract for synthesizing ZnO nanoparticles with specific desired characteristics is a crucial step. Antibiotic properties have been evaluated using both Gram negative organisms (*S. paratyphi*, *Klebsiella planticola*, and *P. aeruginosa*) and Gram-positive organisms (*S. mutans* and *S*. *aureus*) using the well diffusion method.

### 5.2. Magnesium oxide

Magnesium oxide (MgO) nanoparticles have many different fundamental properties that are beneficial for a lot of different applications. For example, MgO is an excellent anti-bacterial agent and is also used as a catalyst, and it is also used in optoelectronic devices and biodiesel synthesis.^213^ It has great corrosion resistance along with high thermal and low electrical conductivity, which allow it to be used in ceramics, additives, photochemical products, and drug manufacturing.^214^ The properties of MgO nanoparticles depend on the fabrication conditions. Different-sized nanoparticles have different calcination reactions and different conditions of gel preparation like pH, heating rate, *etc.*^215^

#### 5.2.1. Green synthesis of MgO nanoparticles

*Lawsonia intermis* leaves were gathered and properly cleaned with deionised (DI) water. Following Akshaykranth *et al.*’s method, the clean leaves were dried and powdered.^216^ 15 g of powdered leaves were taken and put in 150 mL of DI water and heated to 90 °C for 1 h using a hot plate until the solution turned light red. The leaf extract was filtered. Then, the extract was added to 0.5 M Mg(CH_3_COO)_2_ and heated to 80 °C until nanoparticles were formed. The nanoparticles were extracted and calcined at 200 °C for 4 h, and grey-coloured MgO nanoparticles were collected.^216^

For MgO NP synthesis, *Moringa oleifera, Hibiscus rosa sinensis, Citrus limon, Lawsonia intermis* are usually used. The reaction temperature usually ranges from 60 to 90 °C according to different plant extracts used. Table 5 shows that they are most effective against *E*. *coli*, *S*. *aureus*, *B*. *subtilis*, and *P*. *aeruginosa*. Table 5 depicts the various applications of produced MgO nanoparticles using a green method and their potential use as antibacterial agents.

**Table 5:** Formation of MgO nanoparticles from different plant extracts

Raw materials	Plant extract	Reaction conditions	Microbes	Remarks	References
Magnesium acetate	*Lawsonia intermis*	80 °C	*P*. *vulgaris*, *B*. *subtilis*, *S*. *aureus*, and *E*. *coli*	Anti-bacterial	216
Magnesium chloride	*Moringa oleifera*	90 °C, 3 h, stirred	*E*. *coli* and *S*. *aureus*	Anti-bacterial and anti-oxidant	217
	*Bauhinia purpurea*	90 °C, 3 h, stirred	*S. aureus*	Antibacterial	218
Magnesium sulphate	*Hibiscus rosa sinensis*	60 °C, 2 h, stirred pH = 5.6	*P. vulgaris*, *P*. *aeruginosa*, and *E*. *coli*	Antibacterial	219
	*Clematis orientalis*	50 °C, 2 h, stirred	*S*. *aureus*, *B*. *subtilis*, *E*. *coli*, *P*. *aeruginosa*, and *K*. *pneumoniae*	Antimicrobial and antioxidant	220
	*Terminalia bellirica*	78 °C, 4 h, stirred at 110 rpm	*Azotobacter*, *Rhizobium*, and breast cancer cells (MCF-7)	Antioxidant, antibacterial, and antiproliferative	221
Magnesium nitrate	*Persimmon*	60 °C, 2 h, stirred, pH 10–12	*S*. *aureus* and *E*. *coli*	Antibacterial	222
	*Solanum trilobatum*	5000 rpm, 5 min	*E*. *coli*, *B*. *subtilis* and *S*. *pyogenes*	Anti-oxidant and anti-bacterial	223
	*Mucuna pruriens*	RT 10 min, 10 000 rpm	*E*. *coli*, *P*. *aeruginosa*, *S*. *aureus*, and *B*. *subtilis*	Antibacterial and antioxidant	224
	*Citrus limon*	70 °C, 1 h. pH 9.7	*E*. *coli* and *S*. *aureus*	Antimicrobial	225
	*Psidiumguajava* and *Aloe vera*	RT, 30 min, stirred	*S*. *aureus* and *E*. *coli*	Antimicrobial	213
	*Saussurea costus*	80 °C, 4 h, stirred	*S*. *aureus*, *B*. *subtilis*, *P*. *aeruginosa*, *E*. *coli*	Antimicrobial	214
	*Rhizophora lamarckii*	100 °C, 30 min, stirred	*S*. *aureus*, *S*. *pneumoniae*, *E*. *coli*, and *S*. *typhi*	Antimicrobial	226
	*Dalbergia sissoo*	30 °C, 4 h	*E*. *coli* and *Ralstonia solanacearum*	Photocatalytic and antibacterial	227
	*Datura stramonium*	50 °C, 2 h, stirred pH = 2	*E*. *coli* and *S*. *aureus*	Antibacterial	228
	*Punica granatum*	40 °C, 1 h, stirred	*B. subtilis*, *S*. *aureus*, *P*. *aeruginosa*, and *E*. *coli*	Antibacterial and larvicidal	229
	*Abrus precatorius* L.	RT, 45 min, stirred	*Staphylococcus epidermidis*, *B*. *subtilis*, *P*. *aeruginosa*, and *E*. *coli*	Photocatalytic, antioxidant, cytotoxic, and antibacterial	230
	*Emblica officinalis*	RT, 4 h, stirred	*S*. *aureus* and *E*. *coli*	Antibacterial	231
	*Rosa floribunda charisma*	90 °C, 6 h, stirred	*S*. *epidermidis*, *S*. *pyogenes*, and *P*. *aeruginosa*	Antibiofilm, antioxidant, and antiaging	232
	*Limonia acidissima*	RT, 5 min, stirred	*Alternaria alternata*, *Phomopsis azadirachtae*, *P*. *aeruginosa*, *K*. *pneumoniae*, *S*. *aureus*, and *E*. *coli*	Antifungal and antibacterial	233
	*Hagenia abyssinica*	60 °C, 2 h, stirred	*S*. *aureus* and *E*. *coli*	Antibacterial	234
	*Costus pictus*	80 °C, 4 h, stirred	*S*. *aureus*, *B*. *subtilis*, *E*. *coli*, *S*. *paratyphi*, *C*. *albicans*, and *A*. *niger*	Antimicrobial and anticancer	235
	*Citrus aurantium*	80 °C, 2 h, stirred	*S*. *aureus*, *S*. *epidermidis*, *C. albicans, P*. *aeruginosa*, and *K*. *pneumoniae*	Antimicrobial	236

#### 5.2.2. Antimicrobial properties and mechanism of magnesium oxide nanoparticles

Because of their antimicrobial properties, MgO nanoparticles have several applications, such as in water treatment processes, textiles, food packaging, and medicines.^237^ They damage the bacterial cell wall because of the electrostatic interaction between the bacterial cell wall and nanoparticles.^238^ The high alkalinity of MgO also helps damage the bacterial cell wall, producing extreme conditions like a sudden increase in pH that causes the cytoplasm to leak out and lose more fluid, which ultimately causes cell death. A string layer of water forms over the particles when the nanoparticles absorb H_2_O, and this highly alkaline surface of water damages the membrane and kills the bacterial cell.^239^

MgO nanoparticles release Mg^2+^ ions, which interfere with many cellular processes and enzymatic activities.^240^ MgO nanoparticles' high surface energy permits them to adhere to the cell membrane, disrupting its integrity. This causes the cell membrane to rupture and the leaking of cellular components. It has also been shown to disrupt bacterial glycolysis and cause high DNA damage, so the bacteria cannot multiply.^239^

MgO nanoparticles are shown to produce superoxides with the O_2_ from the bacterial cell wall. These superoxides destroy the phospholipids inside the cell wall faster.^241^ Magnesium nanoparticles have extraordinary morphological properties, high surface area, greater stability, and a high refractive index.^242^ It has been shown to be effective in cancer treatment, especially against the leukaemia K562 cell line, as reported by Behzadi *et al.*^243^ MgO nanoparticles also show antifungal activity.^244^ The electrostatic interaction between the negative charge on the fungal cell wall containing glycoprotein and the positive charge of the magnesium oxide nanoparticles causes variation in the zeta potential of the cell barrier and hence reduces membrane strength and stability.^245^

Several methods have been used to synthesize magnesium nanoparticles, for example, thermal decomposition, combustion, chemical precipitation, and sol–gel methods.^246^ However, these methods often need high temperatures and complex reaction conditions like pH control, expensive equipment, and toxic chemicals.^225^ Moreover, because heavy metals like Ti attach to the synthesized nanomaterials, chemical and biological hazards increase in chemical synthesis methods.^226^ Green synthesis of these nanoparticles hence provides a more energy-efficient and economical way with no production of environmentally toxic chemicals. Plants are readily available and biocompatible to use and hence are generally used in the green production of magnesium oxide nanoparticles. Many different phytochemicals are present in these extracts made of plants that cause reduction and act as chemical stabilizers during the formation of magnesium oxide nanoparticles.^247^ Many factors, including pH, plant concentration, or temperature, influence the dimensions and morphology of the MgO nanoparticles formed. For instance, Jeevanandam *et al.* demonstrated that at pH 3, the nanoparticles' size decreases, forming a hexagonal structure.^248^ The disc diffusion technique was used to investigate magnesium oxide nanoparticles' bactericidal action using both Gram negative bacteria (*S. typhi* and *E. coli*) and Gram positive bacteria (*S. pneumoniae* and *S. aureus*).

### 5.3. Cobalt oxide

Cobalt oxide (Co_3_O_4_) nanoparticles are one of the most extensively used nanoparticles nowadays for a number of reasons. Lithium-ion batteries,^249^ gas sensors,^250^ electrochromic thin films,^251^ field emission materials, capacitors,^252^ solar selective absorbers,^253^ catalysis,^254^ and magneto-resistive devices^255^ all use cobalt oxide nanoparticles. They are also used for photocatalytic dye degradation.^256^ The reason for them being successfully used in many applications is that cobalt oxide nanoparticles have magnetic, catalytic, electrical, and optical properties.^257,258^ Many methods can be used to prepare cobalt oxide nanoparticles, like electrochemical, co-precipitation, hydrothermal,^259^ solvent evaporation, and sol–gel processes.^260^ The sol–gel process produces nanoparticles with good chemical reactivity, uniform compound, better efficiency, and good chemical reactivity.^261^ However, these processes may include toxic organic reducing and capping materials or high-temperature calcination, which causes a reduction in the antibacterial properties of the synthesized nanoparticles.^262^ Thus, green synthesis is recommended because it is ecologically safe, non-toxic, and readily accessible in nature, therefore making it inexpensive.^263^

#### 5.3.1. Green synthesis of cobalt oxide nanoparticles

*Fenugreek leaves* were used to generate cobalt oxide nanoparticles by Akhlaghi *et al.*^264^ The leaves were rinsed with distilled water, then dried and powdered. The plant extract was obtained using the ethanolic extraction technique. The extract was mixed with 0.1 M CoCl_2_·6H_2_O in a ratio of 1 : 4. Temperature was maintained at 80 °C using a water bath. The mixture was agitated with a magnetic stirrer until cobalt oxide nanoparticles were formed. Then, it was cooled, filtered, and rinsed with Milli-Q water, and at 13 500 rpm, it was centrifuged three times for 30 min each. At 100 °C, the nanoparticles were then dried for 2 h.^264^

Cobalt oxide has larger incubation times in hours with reaction temperatures from 60 to 90 °C, as shown in Table 6. The data show that *Lawsonia intermis* is most effective against maximum microbes from *A*. *niger*, *A*. *flavus*, *A*. *oryzae*, *Meyerozyma caribbica*, *Meyerozyma guilliermondii*, *R*. *oryzae*, *P*. *aeruginosa*, *Sphingobacterium thalpophilum*, *E*. *coli*, *Sphingobacterium* sp., *S*. *aureus*, *B*. *subtilis*, and *Acinetobacter* sp. to *Ochrobactrum* sp. Table 6 depicts the abundant applications of produced cobalt oxide nanoparticles using a green method and their potential use as antibacterial agents.

**Table 6:** Formation of cobalt oxide nanoparticles from different plant extracts

Raw materials	Plant extract	Reaction conditions	Microbes	Remarks	References
Cobalt(ii) nitrate	*Populous ciliata*	80 °C, 3 h	*E*. *coli*, *K*. *pneumoniae*, *B*. *subtilis*, and *Bacilluslicheniformis*	Antibacterial	265
	*Psidium guajava*	Hot plate, 3 h	*S*. *aureus* and *E*. *coli*	Photocatalytic, antioxidant, antibacterial, and cytotoxic	266
	*Phytolacca dodecandra*	RT, 1 h, stirred	*S*. *aureus* and *E*. *coli*	Antibacterial	267
	*Sesbania sesban*	80 °C, 12 h, stirred	*E*. *coli*, *P*. *aeruginosa*, *S*. *aureus*, and *E*. *faecalis*	Antibacterial	268
	*Lawsonia inermis*	70 °C, 2 h	*A*. *niger*, *A*. *flavus*, *A*. *oryzae*, *M*. *caribbica*, *M*. *guilliermondii*, *R*. *oryzae*, *P*. *aeruginosa*, *S*. *thalpophilum*, *E*. *coli*, *Sphingobacterium* sp., *S*. *aureus*, *B*. *subtilis*, *Acinetobacter* sp., and *Ochrobactrum* sp.	Antifungal and antibacterial	269
	*Ziziphora clinopodioides*	60 °C, 90 min, refluxed	*E*. *coli*, *S*. *typhimurium*, *B*. *subtilis*, and *S*. *pneumoniae*	Cytotoxic, antioxidant, antifungal, and antibacterial	270
	*Curcuma longa*	RT, 30 min, 750 rpm stirred	*S*. *aureus* and *E*. *coli*	Antibacterial, anti-fungal, antimalarial, antiulcer, and antidiabetic	271
Cobalt(ii) chloride	*Ziziphusoxyphylla* Edgew.	80 °C, 30 min	*E*. *coli* and *S*. *aureus*	Antibacterial	272
	*Clitoria ternatea*	RT, 20 min, stirred	*Streptococcus thermophilus*	Antibacterial and antifungal	273
	*Citrus limon*	90 °C, 2 h, stirred at 800 rpm	*S*. *aureus*, *S*. *mutans*, *K*. *pneumoniae*, and *E*. *coli*	Antibacterial and antifungal	274
	*Aerva javanica*	RT, incubated in the dark for 3 days	*B. subtilis*, *S*. *aureus*, *E*. *coli*, and *P*. *aeruginosa*	Antibacterial and antifungal	275
	*Celosia argentea*	80 °C, 30 min, stirred	*E*. *coli* and *B*. *subtilis*	Antioxidant, antibacterial, hemolytic, and catalytic	262
	*Catharanthus roseus*	80 °C, 1 h, stirred	*B*. *subtilis* and *E*. *coli*	Antibacterial, antioxidant, hemolytic, and dye degradation	276
	*Rosmarinus officinalis*	70 °C, 3 h, stirred, pH = 7.5	U87 human cancer cell line, *C*. *albicans*, *E*. *coli*, and *S*. *aureus*	Cytotoxic, antibacterial, and antifungal	277
	*Calpurnia aurea*	60 °C, 2 h, stirred, pH-13	*S*. *aureus* and *E*. *coli*	Antibacterial	278
	*Hibiscus rosa-sinensis*	90 °C, stirred	*S*. *aureus*, *S*. *mutans*, *K*. *pneumoniae*, and *E*. *coli*	Antibacterial and antifungal	279
Cobalt(ii) acetate	*Sageretia thea*	60 °C, 2 h, stirred	*E. coli*	Antibacterial and cytotoxic	280
	*Rhamnus virgata*	75 °C, 2 h, stirred	*A. niger*, *F*. *solani*, *C*. *albicans*, *S*. *aureus*, *E*. *coli*, *K*. *pneumoniae*, *B*. *subtilis*, and *P*. *aeruginosa*	Antibacterial, antileishmanial, antifungal, and anticancer	281
	*Calotropis procera*	RT, 2 h, stirred	*E*. *coli*, *Pseudomonas* sp., *Alcaligens* sp., and *Enterococcus* sp.	Antimicrobial	282
	*Geranium wallichianum*	60 °C, 2 h, stirred	*P*. *aeruginosa*, *B*. *subtilis*, *K*. *pneumoniae*, *E*. *coli*, *S*. *aureus*, *C*. *albicans*, and *A*. *flavus*	Antibacterial, antifungal, anticancer, antioxidant, cytotoxic, and enzyme inhibition	283
Cobalt(iii) sulfate	*Euphorbiatirucalli*	60 °C, overnight, stirred	MCF-7 breast cancer cell	Anti-proliferative	284

#### 5.3.2. Antimicrobial properties and mechanism of cobalt oxide nanoparticles

Cobalt oxide nanoparticles have illustrated excellent antibacterial properties, and they are effective against both Gram negative bacteria (*Pasteurella multocida* and *P. aeruginosa*) and Gram-positive bacteria (*B. subtilis* and *S. aureus*). Cobalt oxide nanoparticles destroy the rigid peptidoglycan layer of Gram-positive bacteria by releasing toxic cobalt ions, which can penetrate the bacterial cell membrane and prevent any protein synthesis. They also cause DNA damage and prevent cell replication. Co_3_O_4_ nanoparticles also damage the membrane and cause all the cellular components to leak out. The ROS generated by cobalt nanoparticles also target both Gram negative and Gram-positive bacteria and cause oxidative stress in bacteria, leading to the degeneration of proteins, lipids, and DNA. Thus, it is used in many medical devices as a coating to prevent bacterial infections.^285^ Adding cobalt nanoparticles to fabrics can create antibacterial textiles, which may be used in healthcare industries. They are also used in water treatment to remove bacteria and in cancer therapy.^286^

Green synthesis can be done through a variety of different natural reducing agents like plant extracts or microorganisms to manufacture nanoparticles with controlled size, shape, and enhanced functionality.^287^ They generally have mild reaction conditions and hence have low energy consumption and less need for complex instruments.^288^ They are also environmentally friendly, helping us avoid the use of toxic organic solvents.^289^ Nanoparticles made from toxic chemicals may retain some residual impurities, which will affect their biocompatibility. Green synthesis methods can also be easily scaled up for industrial production and are a great cost-effective alternative.^290^ Also, traditional nanoparticle synthesis can generate a lot of harmful waste. These wastes need to be disposed safely to prevent environmental pollution. Nanoparticles synthesized using green methods are often more biocompatible and thus can be applied in biomedical industries for delivering drugs and as antibacterial agents.^263^ Studies have shown that using plant extracts like *Curcuma longa* or *Aloe vera* can effectively reduce metal ions to nanoparticles. These extracts contain phytochemicals, which can be both stabilizing and reducing agents, so there is no need for harmful chemicals.

### 5.4. Titanium dioxide

Titanium dioxide nanoparticles are promising materials for antimicrobial application. They are a widely studied form of nanomaterial with unique properties, which make it suitable for many applications. They are used in sunscreens as they can block UV radiation while remaining transparent on the skin.^291^ They are also used in paints, coatings, and food packaging.^292,293^ These nanoparticles are also used in the construction of buildings and in cement, tiles, plastics, windows, solar cells, and also in light-emitting diodes.^291^

#### 5.4.1. Green synthesis of titanium dioxide nanoparticles

To synthesize TiO_2_ nanoparticles from titanium isopropoxide, *Syzygium cumini* leaves were used as a capping agent, as suggested by Sethy *et al.*^294^ The leaves are collected and rinsed carefully with DI water and dried at 60 °C in a tray drier. Then, they were ground into powder, and made into a solution with DI water in a 20 : 100 ratio. At 80 °C, it was heated for 1 h. The plant extract was filtered and combined with titanium isopropoxide at a ratio of 1 : 1. The mixture was agitated for 8 h. At 9000 rpm, it was centrifuged for 10 min and filtered. The nanoparticles formed were then calcined at 570 °C for 3 h.^294^

Many different plant extracts were found to successfully produce titanium dioxide nanoparticles with antibacterial, antioxidant or photocatalytic activities such as *Calotropis procera*, *Acorus calamus*, *Maerua oblongifolia*, and *Chenopodium quinoa* with being *Trigonella foenum* effective against the highest number of microbes such as *S*. *aureus*, *E*. *faecalis*, *K*. *pneumoniae*, *S*. *faecalis*, *P*. *aeruginosa*, *E*. *coli*, *P*. *vulgaris*, *B*. *subtilis*, *Yersinia enterocolitica*, and *C*. *albicans*, as shown in Table 7. The reaction temperature ranged from RT to 80 °C with vigorous stirring. Table 7 shows various applications of produced TiO_2_ nanoparticles using a green method and their potential use as antibacterial agents.

**Table 7:** Formation of TiO_2_ nanoparticles from different plant extracts

Raw materials	Plant extract	Reaction conditions	Microbes	Remarks	References
Titanium tetraisopropoxide	*Calotropis procera*	RT, 2 h, stirred	*S*. *aureus*, *Streptococcus* sp., *E*. *coli*, and *P*. *aeruginosa*	Antibacterial and antioxidant	295
	*Acorus calamus*	RT, stirred, pH = 7	*S*. *aureus*, *P*. *aeruginosa*, *E*. *coli*, and *B*. *subtilis*	Antimicrobial and photocatalytic	296
	*Nervilaaragona*	RT, stirred	*E*. *coli* and *S*. *aureus*	Antibacterial	297
	*Menthe arvensis*	50 °C, 5 h, stirred	*E*. *coli*, *P*. *vulgaris*, and *S*. *aureus*	Antibacterial and antifungal	298
	*Maerua oblongifolia*	60 °C, 1 h, stirred	*E*. *coli* and *S*. *aureus*	Antibacterial and photocatalytic degradation	299
	*Chenopodiumquinoa*	RT, stirred	*Ustilago tritici*	Antifungal	300
Titanium sulfate	*Luffa acutangula*	RT, 6 h, stirred	*B. subtilis*, *E*. *coli*, *K*. *pneumoniae*, *E*. *faecalis*, and *S*. *aureus*	Antibacterial, antifungal, and optical	301
Titanium oxysulfate	*Acacia catechu*	80 °C, 2 h, stirred	*S. aureus*	Antibacterial	302
	*Ocimum americanum*	60 °C, 30 min, stirred	*B*. *cereus*, *Clostridium perfringens*, *K*. *pneumoniae*, *S*. *paratyphi*, *C*. *albicans*, and *A*. *niger*	Photocatalytic and antimicrobial	303
	*Trigonella foenum*	RT, 15 min, stirred	*S*. *aureus*, *E*. *faecalis*, *K*. *pneumoniae*, *S*. *faecalis*, *P*. *aeruginosa*, *E*. *coli*, *P*. *vulgaris*, *B*. *subtilis*, *Y*. *enterocolitica*, and *C*. *albicans*	Antimicrobial	304
Titanium oxyhydroxide	*Cola nitida*	RT, 1 h	*S*. *aureus*, *P*. *aeruginosa*, *K*. *pneumoniae*, and *E*. *coli*	Antibacterial, antifungal, antioxidant, and anticoagulant	305
	*Mangifera indica*	RT, 24 h, stirred	*Asubpictus* and *Culex quinquefasciatus*	Larvicidal	306
	*Artemisia haussknechtii*	RT, 24 h, stirred	*S*. *aureus*, *S*. *epidermidis*, *Serratia marcescens*, and *E*. *coli*	Antibacterial and antioxidant	307
	*Psidium guajava*	RT, 24 h, stirred	*A. hydrophila*, *P*. *mirabilis*, *E*. *coli*, *S*. *aureus*, and *P*. *aeruginosa*	Antioxidant and antibacterial	308
Titanium tetrabutoxide	*Kniphofia foliosa*	RT, 4.5 h, stirred	*S*. *aureus*, *S*. *pyogenes*, *E*. *coli*, and *K*. *pneumoniae*	Antibacterial	309
	*Citrus limon*	RT, stirred	*E. coli,* methicillin-resistant *S. aureus,* and*Klebsiella* sp.	Antibacterial	310
Titanium dioxide	*Azadirachta indica*	RT	*S*. *typhi*, *E*. *coli*, *S*. *aureus*, *K*. *pneumoniae*, and *B*. *subtilis*	Antibacterial	311
	*Allium eriophyllum* Boiss	RT, stirred	*S*. *pneumoniae*, *S*. *typhimurium*, *C*. *glabrata*, *C*. *albicans*, *C*. *guilliermondii*, *S*. *aureus*, and *P*. *aeruginosa*	Antioxidant, cytotoxic, antibacterial, and antifungal	291
	*Allium cepa*	50 °C, 4–5 h, stirred at 1000 rpm	*S*. *aureus*, *K*. *pneumoniae*, *S*. *epidermidis*, *E*. *coli*, and *C*. *albicans*	Antimicrobial	312
	*Andrographis paniculata*	RT, 1 h, stirred	*E*. *coli*, *Salmonella* sp., *Bacillus* sp., and *C. albicans*	Antioxidant, antidiabetic, and antibacterial	313
Titanium(iv) chloride	*Citrullus lanatus*	0 °C, 3 h, stirred, microwave (1000 W) 3 min	*C*. *neoformans*, *B*. *subtilis*, *E*. *coli*, *C*. *albicans*, *Aspergillus fumigatus*, and *A*. *niger*	Antioxidant, cytotoxic, and antimicrobial	314
	*Aloe barbadensis* Mill.	RT, stirred, pH = 7	*P. aeruginosa*	Antibacterial RT	315

#### 5.4.2. Antimicrobial properties and mechanism of titanium oxide nanoparticles

TiO_2_ is known for its antimicrobial and strong photocatalytic properties; thus, it is very useful in medical applications.^316^ Rajeswari *et al.* showed that these nanoparticles have strong antioxidant properties, even better than ascorbic acid.^317^ They can generate ROS when exposed to light which cause oxidative damage to microbial cells and also tumour cells.^318^ The excited electrons participate in redox reactions with water and oxygen molecules, forming highly reactive species:h^+^ +H_2_O → ˙OH + H^+^e^−^ + O_2_ → ˙O_2_^−^

These ROS can cause oxidative damage to a broad range of biological molecules like proteins and DNA, causing cell death. TiO_2_ nanoparticles are effective against a large spectrum of bacteria. They are used as a coating in many medical instruments to prevent the growth of microorganisms.^319^ In cancer treatment, TiO_2_ nanoparticles are used in photodynamic therapy (PDT), where they are triggered by light to create ROS, which selectively destroy cancer cells.^320^ The ease of surface modification in these nanoparticles makes them easier to use in nanocarriers for targeted drug delivery.^321^ Also, titanium dioxide nanoparticles are shown to release drugs in response to specific stimuli like changes in pH or temperature. Thus, they are effective in drug delivery where they release ROS in the presence of an acidic environment due to the tumour but do not affect the neutral healthy cells. Achudhan *et al.* conducted a study demonstrating the mosquito-killing effects of titanium dioxide nanoparticles synthesized using a solution from *Ficus benghalensis*, *Azadirachta indica twigs*, and *Syzygium aromaticum*.^322^ According to the research, these nanoparticles successfully targeted *Aedes aegypti larvae*, causing damage to the thoracic and abdominal regions as well as body shrinkage and hair bristle loss.

Many synthesis processes are used to synthesize metal oxide nanoparticles, such as the electrochemical method, the sonochemical methodology, or the sol–gel process.^323,324^ TiO_2_ nanoparticles can be synthesized using fungi, bacteria, and plant extracts as capping and reducing agents.^325^ This green synthesis method offers an affordable and natural alternative to conventional chemical production methods. Not only is it renewable and non-toxic but it also reduces energy consumption. Furthermore, the green synthesis method aligns with the global push to circular economy models, where waste is minimized, and resources are efficiently utilized.

## 6. Antimicrobial properties of metal-based nanoparticles

Numerous processes, such as metal ion release, oxidative stress induction, and non-oxidative damage, which impact various microorganisms' structures, have been clarified as part of the extensive investigation into the precise antibacterial mechanisms of NPs.^326^

### 6.1. Reactive oxygen species (ROS) production

**Fig. 4:**
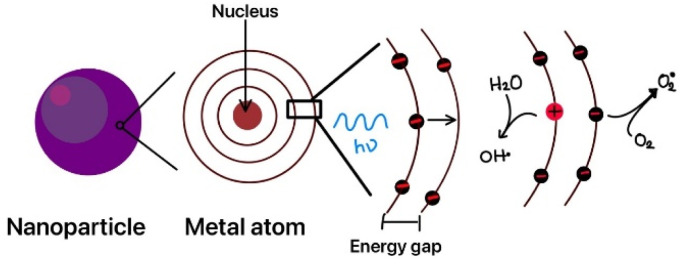
ROS production by metal nanoparticles.

Reactive oxygen species are short-lived reactive intermediates or molecules (with half-lives ranging from 10^−9^ to 10^−3^ seconds) that possess significant oxidative potential, which can ultimately be toxic to microorganisms.^327^ They function in cell signaling and homeostasis at low concentrations and induce oxidative stress and damage at elevated concentrations. When metal compounds absorb sufficient energy from light irradiation, an electron is excited and moves to the conduction band from the valence band, creating a highly reactive hole (H^+^). The holes in the valence band show high oxidizing power, whereas the electrons in the conduction band demonstrate high reducing power. This reactive site interacts with the surrounding H_2_O or OH^−^ molecules, generating reactive oxygen species (ROS) near the nanoparticles, as shown in Fig. 4.^328^ Nanoparticles can also produce ROS through Fenton and Haber–Weiss reactions in which metal ions such as Fe^2+^ react with hydrogen peroxide and generate hydroxyl radicals.^329^Fe^2+^+ H_2_O_2_ → Fe^3+^+ ˙OH + OH^−^

The Haber–Weiss reaction continues ROS formation by recycling ferric iron:O^2−^ + H_2_O_2_ → O_2_+ ˙OH + OH^−^

Another method of producing ROS is metal nanoparticles coming in contact with biological molecules and causing them to produce ROS through redox cycling reactions. Silver and gold nanoparticles are metabolically active in bacteria and cause oxidative stress.^330^ ZnO and TiO_2_ NPs are shown to exhibit three different types of ROS under UV exposure – singlet oxygen, superoxide radicals, and hydroxyl radicals. Some metal oxides produce only one or two different radicals.^331^ Thus they are mostly used in photodynamic therapy. The hydroxyl radical is a nonselective and highly reactive oxidant that damages every kind of biomolecule like lipids, DNA, nucleic acids, proteins, amino acids, and carbohydrates. Metal nanoparticles (NPs) utilize reactive oxygen species (ROS) and reactive nitrogen species (RNS), including peroxynitrite (ONOO^−^) and nitric oxide (NO), to exert antimicrobial effects. ROS, such as superoxide and hydrogen peroxide, can damage bacterial DNA, proteins, and membranes, leading to cell death.^332^ Nanoparticles that produce ROS have been explored as adjuvants to conventional antibiotics. They enhance antibiotics by disrupting bacterial cell membranes and DNA and the cellular functions. This synergistic approach can be used against various drugs and strains of bacteria. NPs can enhance ROS production through various mechanisms, including Fenton reactions and photodynamic therapy, which increase their antimicrobial efficacy.^333,334^ RNS also play a role in modulating microbial responses to oxidative stress, potentially enhancing the effectiveness of metal NPs in combating infections, and also causes oxidative protein carbonylation, inactivation of specific enzymes, as well as lipid peroxidation and nitrosative stress.^335^ ROS can initiate the peroxidation of polyunsaturated fatty acids in membranes which damage membrane integrity and function. Brynildsen *et al.* have deployed a system biology approach aimed at identification of metabolic reactions in *E. coli* that generates ROS. They discovered that removing genes for metabolic enzymes made bacteria more sensitive to β-lactam and fluoroquinolone antibiotics. Blocking the action of succinate dehydrogenase also increased sensitivity to oxidants and ampicillin. This suggests that increasing endogenous ROS locally can help improve the activity of antibiotics.^336^

This dual action of ROS and RNS highlights the therapeutic potential of metal NPs in antimicrobial applications. Due to their ability to produce ROS, nanoparticles like Ag, CuO, Au and ZnO are included in medicinal coatings to avoid colonization of bacteria on implants and surgical tools. These coatings ensure a sustained release of ROS over time without an antibiotic to kill microbes. Using nanoparticles that create ROS is a new form of antimicrobial therapy. Future studies should work to improve the formulation of nanoparticles for better selection, stability and less toxicity, which will help the next-generation antimicrobial treatments.^337^

#### 6.1.1. Cell wall damage

The composition of the cell wall varies depending on the type of microorganism: Gram-positive bacteria have teichoic acid (20–50 nm) and multiple layers of peptidoglycan; fungi and yeast are primarily made of chitin and polysaccharides; Gram-negative bacteria have less layers of peptidoglycan encircled using another lipid membrane that contains lipoproteins and lipopolysaccharides.^338^

Because of the features of teichoic acid structures and peptidoglycan, the attraction of the nanoparticles with the walls is greater, which causes cell membrane damage and cell death; the cell wall has a higher negative charge than Gram-negative bacteria.^339^ Through electrostatic attraction, hydrophobic interactions, and van der Waals forces, zinc oxide, silver, titanium dioxide, and gold nanoparticles can be drawn to the cell wall, altering the permeability, shape, and function of the cells.^340–342^

#### 6.1.2. Proteins of DNA

**Fig. 5:**
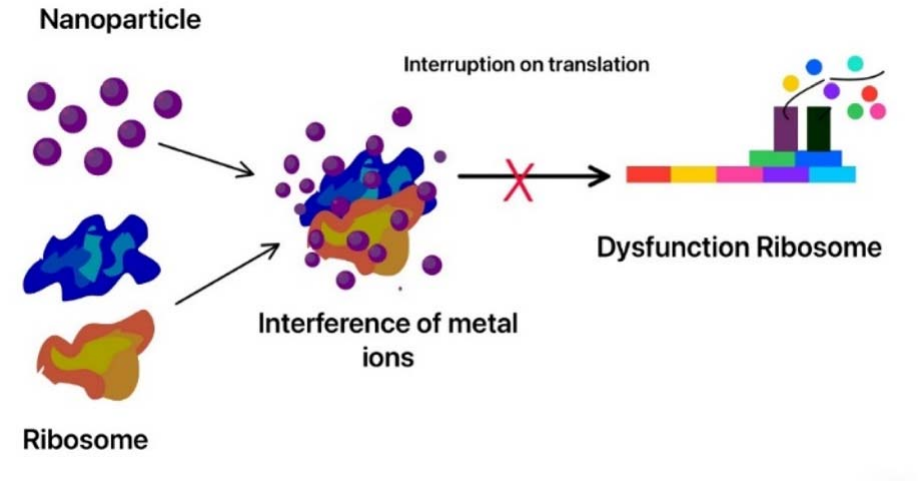
Damaging ribosomal functions of micro-organisms using metal nanoparticles.

**Fig. 6:**
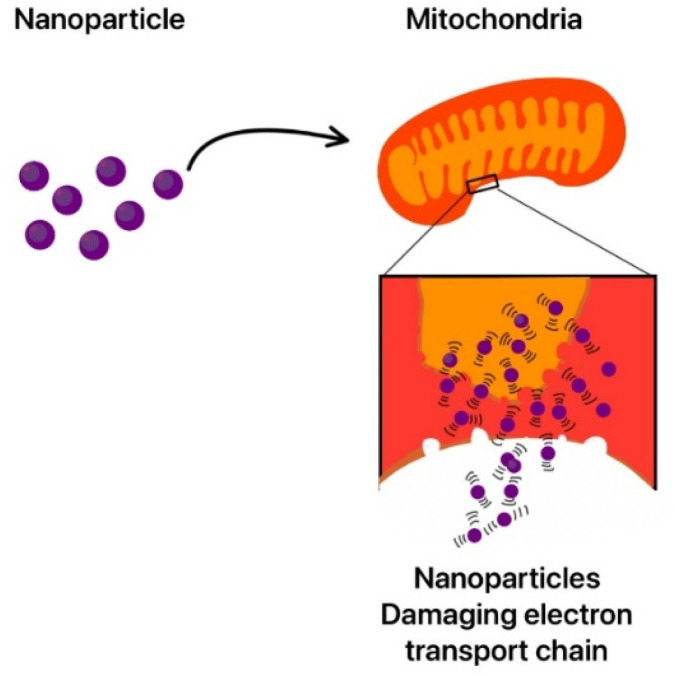
Damaged electron transport chain in mitochondria of bacteria using metal nanoparticles.

Proteins are an essential component of cellular structures and play a key role in metabolic reactions that microorganisms catalyze. In the presence of CuO NPs, proteomic analysis has shown deregulation in proteins present in substance transport, nitrogen metabolism, and electron transfer.^343^ Even in bacteria with cell walls and plasma membranes, the expression of the ribosomal subunit, which binds with protein groups that contain phosphorus and sulfur, can be impacted by the silver ions released by Ag NPs.^344^ Au NPs inhibit ATPase activity by collapsing the membrane potential and preventing a ribosomal subunit from combining with tRNA, as shown in Fig. 5. This consequently decreases the ATP levels and increases ROS production, impacting other structures at the same time, as illustrated in Fig. 6.^343^

**Fig. 7:**
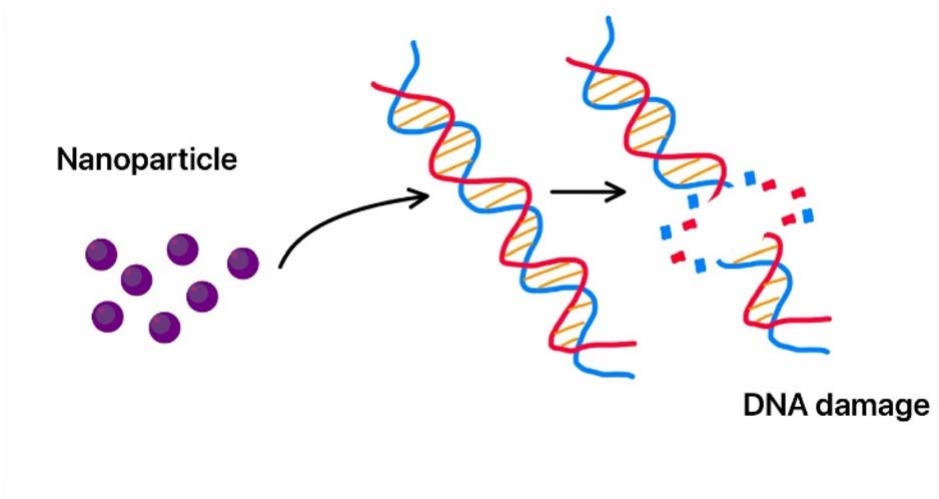
Damaging DNA structure of bacteria utilizing metal nanoparticles.

Since ROS can cause DNA mutations, genomic analysis has demonstrated that TiO_2_ NPs can impact transcription, cell division, and metabolic replication in regulatory microorganisms. Saccharide fragmentation and strand break may result from these changes, which may attack the sugar-phosphate or the nucleobases.^339^ Using electrophoresis, the cleavage caused by the nanoparticles was examined in the pBR322 plasmid in the presence of Ag NPs.^345^ Guanine's low redox potential makes it the most affected nucleobase, according to the results, and its oxidation results in a wide range of changes that eventually impact DNA function (Fig. 7).^346^ By inducing genetic damage, nanoparticles can impact not only bacteria but also other intricate multicellular organisms.^347^ Copper oxide nanoparticles (CuONPs) exhibit significant antibacterial properties, primarily through mechanisms that damage bacterial DNA.^348^ They induce oxidative stress, leading to the generation of reactive oxygen species (ROS) that can cause DNA strand breaking and other forms of genetic damage.^349,350^ CuONPs also disrupt bacterial cell membranes and interfere with cellular functions, contributing to their bactericidal effects.^351,352^ Their ability to release copper ions enhances their efficacy, as these ions can bind to essential proteins in bacteria, further promoting cell death.^353^

## 7. Minimizing the obstacles and limitations of green synthesis process

**Fig. 8:**
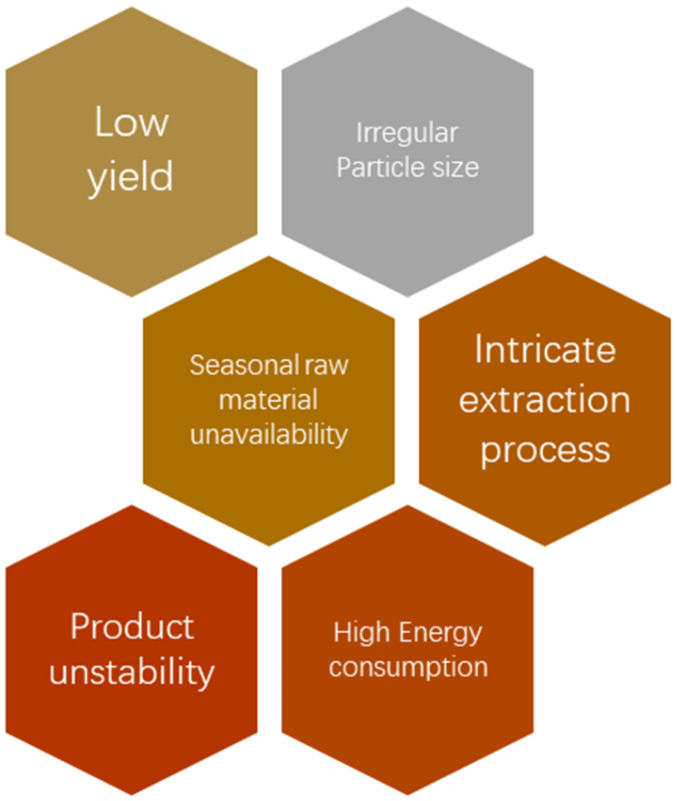
Obstacles of green synthesized metal nanoparticles.

The practical production and use of green synthesized nanomaterials, however, require overcoming a number of obstacles, including low yield, intricate extraction processes, regional and seasonal raw material availability, irregular particle sizes, and other issues illustrated in Fig. 8. Consequently, there may be a wide range of opportunities, as shown in Fig. 1, and significant development potential for green synthesis of nanoscale metals.^354^

**Fig. 9:**
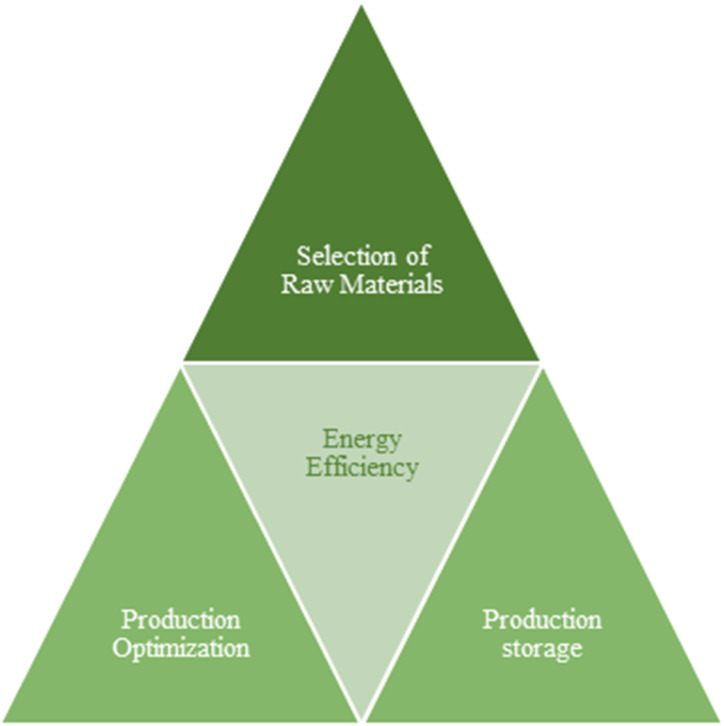
Points to consider to overcome the limitations of the green synthesis process.

The following hypotheses and presumptions need to be considered to get over the flaws and restrictions in the existing research and, ultimately, implement green-synthesized (GS) nanoscale metals (Fig. 9).

Selection of raw materials: evergreen plants are more promising and effective for the selection of plants for metal synthesis in green chemistry. The primary focus of future research should be on materials that are not restricted by geographical or seasonal availability. Before choosing materials, researchers should think about the ease of the extraction process and the seasonal and geographic availability for material acquisition.^354^ An excellent substitute material might be evergreen plants like *Filicium decipiens*, which are able to evade seasonal time constraints. *F. decipiens* trees are frequently used as shade trees, and their growth is typically unrestricted by the soil. Since it was directly acquired from the campus, *F. decipiens* used was easily accessible.^355^ Iron oxide nanoparticles have also been produced using different plants, including *S. lavandulifolia*, *Mediterranean cypress* (*Cupressus sempervirens*), and *Murraya koenigii*.^356,357^ These plant materials have high viability for green synthesis in terms of availability because their collections were not restricted by time or season. Asia, North Africa, Europe, and America are all home to *Urtica dioica*, more commonly known as common nettle or stinging nettle, which is used to synthesize Fe NPs. Grape and eucalyptus leaves are also frequently used to produce Cu NPs.

Optimized production: the creation of uniform small particles with a large surface area should be the main goal of future research. *Gloriosa superba* L. extract is better for CuO NPs since it produces particles with consistent sizes between 5 and 10 nm.^358^ Mint (*Mentha spicata* L.) leaf extract was produced in a uniform small amount for NZVIs. Scattered particles that did not come together during the synthesis of *Cupressus sempervirens* leaf extract have a diameter of roughly 1.5 nm.^359^ Certain eco-friendly materials can be synthesized.

Energy efficiency: based on the current energy-saving experiments, processes that can be performed without heating can be implemented to overcome the problem of high energy consumption. Fe NPs can be produced with leaf extracts from *Henna*, *Gardenia*, and *Hippophae rhamnoides* without the use of hazardous chemical reagents or high reaction temperatures or energies. Some low-energy extraction techniques are presently in use, such as processes utilizing the grape seed extract that produces Ag NPs at temperatures below 100 °C^360^ or fruit extract from *Genipa americana* that produces Au NPs at temperatures between 22 and 25 °C. 80 °C is the temperature necessary for the synthesis of Fe NPs.^361^ Methods with relatively low energy consumption should be taken into consideration when compared to those that require ≥600 °C.

Production storage: nanoscale metal storage must also be considered. The cost of storage or preservation will be significantly reduced if nanoscale metals can be stored at room temperature. The stability of nanoscale metals affects how they are stored. The NPs can be stored more affordably and are more stable. Ag NPs made from *Acorous calamus* rhizome stayed stable for 24 hours, but those made from *Carissa carandas* fruits only displayed the highest UV-vis absorption peak for 4 hours before declining over time. This suggested that after four hours, its stability would be impacted.^362,363^ Cu NPs produced using the leaf extract of *Thymus vulgaris* L. and supported by bentonite can be kept in an inert environment for 20 days.^364^ For 15 days, the Cu NPs that were created using the aqueous guava extract stayed stable at room temperature. PEG 6000 was chosen as a capping agent to keep the metal colloid stable during the 15 days storage period because the copper nanoparticles are oxidized to copper oxide easily.^365^ The product stability can be impacted by various manufacturing techniques and materials. To create more stable products, researchers should select the right materials and techniques.

Some raw materials also need further processing that adds to the intricacy and cost of the green synthesis technique. To prove economic viability, the usefulness and cost-effectiveness of these substances need to be demonstrated. While the local supply of green NPs can be done using native plants, it is difficult to source and use plants globally for nanoparticle synthesis. Nanoparticles' shape and size vary with different plant extracts, and the large difference in the size of the particles make green technology unfit for bulk production; besides, controlling the particle size during production presents a major challenge. Also, the specific chemical pathways involved in the synthesis process are not completely understood even when the effect of plant extracts on synthesis has been established. Phytochemicals help increase the dispersion as well as reduce agglomeration. The properties of phytochemicals found in plant extracts and species used to manufacture nanoformulations play a key role in green synthesis.

## 8. Conclusion

This review highlights the benefits of green synthesis using plant extracts in place of chemical synthesis techniques that are characteristically hazardous and energetically expensive. Plant extracts have been found to produce different shaped and sized nanoparticles with antibacterial, antifungal, cytotoxic, and catalytic properties due to the presence of phenolic acid, alkaloids, terpenoids, and polyphenols which act as reducing and stabilizing agents to successfully synthesize these metal and metal oxide nanoparticles. Thus, different plant extracts can be used to produce nanoparticles with distinct properties for specific purposes. Green synthesis has a bright future because it fits in with the global focus on environmental preservation, sustainability, reliability and technological innovation. Because of its cost-effectiveness, eco-friendliness, and capacity to produce functional nanomaterials with a broad range of applications, green synthesis of nanoparticles, in particular, is expected to expand dramatically across numerous industries. By combining the unique properties of nanoparticles along with the benefits of green synthesis, it aims to address the pressing challenges in healthcare and environmental sustainability while advancing the field of nanotechnology. This sustainable and environmental friendly method of nanoparticle synthesis is extensively utilized in electronics, medicine, catalysis, and environmental applications. This technique avoids the use of hazardous chemicals by utilizing natural reducing agents derived from biological sources. Even though green synthesis has many benefits, issues like reproducibility, scalability, and managing the shape and size of nanoparticles require further study. Both the shape and average size of the metallic nanoparticles have profound effects on their biological and therapeutic activities.^366^ The ongoing research in this field aims to improve techniques and increase their use in industry and medicine. These nanoparticles are highly relevant for the ongoing issue of drug-resistant infections because of their relatively broad-spectrum antibacterial, antifungal, and antiviral activities against a variety of bacteria, fungi, viruses, and drug-resistant pathogens. Different elements of the plants can be used to form numerous metal or metal oxide nanoparticles. Some problems, like adverse reactions, biodegradation, cellular interactions, abundance of raw materials, and biodistribution, need to be considered. Also, researchers must be concerned about not leaving these nanoparticles inside biological systems, where they can accumulate in our food chain and lead to numerous health issues. Using metal NPs like silver nanoparticles in different industries for a prolonged time has been shown to cause severe toxic effects on human health like bluish-grey skin and eye discolouration.^367^ Thus, extensive research is required to enhance production and application in many other fields.

The green synthesis of metal nanoparticles (MNPs) for antibacterial applications faces several limitations:

(1) Reproducibility: achieving consistent particle size and shape can be challenging, affecting the reliability of antimicrobial efficacy.^24,368^

(2) Biological effects: the biological implications of green-synthesized MNPs, including toxicity and biocompatibility, are not well understood, necessitating further research.^369^

(3) Synthesis complexity: the process often involves complex steps such as microbial isolation and culturing, which can complicate production scale and efficiency.^368^

(4) Environmental impact: while greener than traditional methods, the environmental benefits of green synthesis need a thorough evaluation to ensure no harmful by-products are produced.^24,370^

## Data availability

No primary research results, software or code have been included and no new data were generated or analysed as part of this review.

## Author contributions

Israt Jahan Lithi: data curation, formal analysis, writing – original draft. Kazi Imtiaz Ahmed Nakib: formal analysis, writing – original draft, visualization. Md. Sahadat Hossain: conceptualization, supervision, writing – review and editing. A. M. Sarwaruddin Chowdhury: supervision, writing – review and editing.

## Conflicts of interest

The authors declare no financial or personal interests that may have influenced the work presented in this study.
